# Genome-Wide Identification of *Dickeya solani* Transcriptional Units Up-Regulated in Response to Plant Tissues From a Crop-Host *Solanum tuberosum* and a Weed-Host *Solanum dulcamara*

**DOI:** 10.3389/fpls.2020.580330

**Published:** 2020-09-02

**Authors:** Robert Czajkowski, Jakub Fikowicz-Krosko, Tomasz Maciag, Lukasz Rabalski, Paulina Czaplewska, Sylwia Jafra, Malwina Richert, Marta Krychowiak-Maśnicka, Nicole Hugouvieux-Cotte-Pattat

**Affiliations:** ^1^Division of Biologically Active Compounds, Intercollegiate Faculty of Biotechnology UG and MUG, University of Gdansk, Gdansk, Poland; ^2^Division of Biological Plant Protection, Intercollegiate Faculty of Biotechnology UG and MUG, University of Gdansk, Gdansk, Poland; ^3^Division of Recombinant Vaccines, Intercollegiate Faculty of Biotechnology UG and MUG, University of Gdansk, Gdansk, Poland; ^4^Laboratory of Mass Spectrometry - Core Facility Laboratories, Intercollegiate Faculty of Biotechnology UG and MUG, University of Gdansk, Gdansk, Poland; ^5^Laboratory of Electron Microscopy, Faculty of Biology, University of Gdansk, Gdansk, Poland; ^6^Microbiology Adaptation and Pathogenesis, CNRS UMR5240, University of Lyon, University Claude Bernard Lyon 1, INSA Lyon, Villeurbanne, France

**Keywords:** potato, bittersweet nightshade, Tn5, mutagenesis, alternative plant host, *Erwinia chrysanthemi*

## Abstract

*Dickeya solani* is a Gram-negative bacterium able to cause disease symptoms on a variety of crop and ornamental plants worldwide. Weeds including *Solanum dulcamara* (bittersweet nightshade) growing near agricultural fields have been reported to support populations of soft rot bacteria in natural settings. However, little is known about the specific interaction of *D. solani* with such weed plants that may contribute to its success as an agricultural pathogen. The aim of this work was to assess the interaction of *D. solani* with its crop plant (*Solanum tuberosum*) and an alternative (*S. dulcamara*) host plant. From a collection of 10,000 Tn5 transposon mutants of *D. solani* IPO2222 carrying an inducible, promotorless *gusA* reporter gene, 210 were identified that exhibited plant tissue-dependent expression of the gene/operon into which the Tn5 insertion had occurred. Thirteen Tn5 mutants exhibiting the greatest plant tissue induction of such transcriptional units in *S. tuberosum* or *S. dulcamara* as measured by qRT-PCR were assessed for plant host colonization, virulence, and ability to macerate plant tissue, as well as phenotypes likely to contribute to the ecological fitness of *D. solani*, including growth rate, carbon and nitrogen source utilization, motility, chemotaxis toward plant extracts, biofilm formation, growth under anaerobic conditions and quorum sensing. These 13 transcriptional units encode proteins involved in bacterial interactions with plants, with functions linked to cell envelope structure, chemotaxis and carbon metabolism. The selected 13 genes/operons were differentially expressed in, and thus contributed preferentially to *D. solani* fitness in potato and/or *S. dulcamara* stem, leaf, and root tissues.

## Introduction

Soft Rot *Pectobacteriaceae* (SRP: *Pectobacterium* spp. and *Dickeya* spp.) are important, broad host range phytopathogenic, necrotrophic bacteria that cause substantial and increasing economic losses in agricultural crops worldwide (Toth et al., 2003; Charkowski, 2018). In potato, these bacteria are responsible for blackleg and aerial stem rot of plants in the field as well as for tuber soft rot in transit and storage (Pérombelon, 2002).

Although the predominant source of inoculum for soft rot and blackleg bacteria on potato are (latently) contaminated potato tubers (Pérombelon, 2002; Charkowski, 2018), other routes of SRP transmission in environment also contribute to the disease epidemiology (Perombelon and Kelman, 1980; Charkowski, 2007). Indeed, a relatively high incidence of SRP-caused diseases is observed in potato fields even after planting pathogen-free seed tubers (Toth et al., 2011). In these cases, the rapid development of blackleg and soft rot symptoms cannot be easily explained by latent infections of planting material (van der Wolf and de Boer, 2007). Such observations suggest that SRP may overwinter in soil in association with various plants, primarily in protected locations in the roots of alternative host plants and/or on or within volunteer crop plants from previous plantings (Pérombelon, 1992).

Despite the general interest in elucidating the role of alternative hosts in the ecology of soft rot bacteria, little is known about those plant species that could support survival of SRP as well as the multiplication of these bacteria in the absence of potato (Toth et al., 2011). Only a few reports have been published that address such alternative hosts of SRP. For example, Tsror and co-workers described latent infection of *Cyperus rotundus* (nut grass; common weed in Israel) by *Dickeya solani* as well as latent infection of *Malva nicaeensis* with *Pectobacterium brasiliensis* (Tsror et al., 2010; Tsror et al., 2019). *D. solani* was also reported to be able to colonize some common weeds grown in the European climate zone, such as *Urtica urens* (annual nettle) and *Viola arvensis* (field pansy) after artificial infestation of the soil with the pathogen. However, it has not been shown that those plant species are natural environmental reservoirs for SPR or that these latently infected plants can disseminate bacteria to agricultural crops (Elphinstone et al., 2013). Similarly, *Dickeya* spp. (previously *Erwinia chrysanthemi*) were isolated from symptomless *Solanum dulcamara* (bittersweet nightshade) grown in temperate watercourses located near agricultural fields in Sweden (Olsson, 1985). Isolations of SRP from *S. dulcamara* collected near agricultural fields were also reported in the Netherlands (van Vuurde and de Vries, 1992), Finland (Laurila et al., 2006), Australia (Cother and Gilbert, 1990), and recently in Poland (Fikowicz-Krosko et al., 2017).

Irrespective of the relatively high SRP incidence in alternative hosts in natural settings, knowledge of the infection process in plant species other than potato is almost entirely absent (Reverchon et al., 2016; Fikowicz-Krosko and Czajkowski, 2017b). It is noteworthy that the great majority of SRP reports on alternative hosts have been on symptomless plants. Likewise, we have recently shown that although *S. dulcamara* grown under disease-favoring conditions can be systemically infected with *D. solani* after its delivery to stems by stab-inoculation at high densities, the inoculated plants do not develop symptoms (Fikowicz-Krosko and Czajkowski, 2017b).

*D. solani* (van der Wolf et al., 2014) has become one of the major SRP pathogens in (seed) potato cultivation in Europe at the beginning of 2000s (van der Wolf et al., 2017). This pathogen is a distinct genetic clade of biovar 3, causing blackleg and soft rot, characterized after an outbreak in potato plants in Finland (Laurila et al., 2008) and soon after in many European countries (Czajkowski et al., 2009; Slawiak et al., 2009). *D. solani* appears to cause high disease incidence and severity in potato as well as other crops including ornamental plants in both subtropical and temperate climates (Degefu et al., 2013; Tsror et al., 2013). It is believed that the pathogen has been only recently introduced to the potato ecosystem in Europe as most *D. solani* strains show a low level of genetic variations (Khayi et al., 2015).

The purpose of this study was to identify and characterize those *D. solani* transcriptional units (genes and operons) encoding factors contributing to *in planta* fitness and virulence that are: (i) expressed exclusively during plant infection and (ii) expressed in one but not in the other host. To find host-dependent virulence and fitness genes/operons, we examined two plant models: *S. tuberosum*—the model crop host and *S. dulcamara*—a weed model representing an alternative/non-crop host. This strategy was used to help explain the apparent resistance of this later plant to infection caused by *D. solani*. To understand the interaction of *D. solani* with its plant hosts during early stages of interaction we employed a random mutagenesis approach of *D. solani* strain IPO2222 using Tn5 harboring a promotorless *gusA* reporter gene. This system relies on the Tn5-*gusA* transposons that fuse target operons or genes with the promotorless reporter *gusA*. Expression of the reporter gene occurs only when the expression of the gene/operon carrying Tn5-*gusA* is activated. To identify plant host-specific fitness and virulence transcriptional units, we applied a stringent gene selection protocol in which all genes/operons that were expressed in the absence of plant tissues were removed from further analysis in an initial step. This allowed us to reveal bacterial genes/operons expressed solely in one or the other host plant and will enable subsequent studies of their differential contribution to host-specific virulence and ecological fitness in various settings.

## Materials and Methods

### Bacterial Strains, Plants, and Growth Media

The *D. solani* Tn5 mutants characterized in this study are listed in **Table 1**. *Escherichia coli* strain S17 λ-pir with plasmid pFAJ1819 (Xi et al., 1999) (obtained from Belgian Co-ordinated Collections of Microorganisms - BCCM, Brussels, Belgium) was cultured as previously described (Lisicka et al., 2018). Plasmid pFAJ1819 harbors a mini-Tn5 transposon that contains a promotorless *gusA* reporter gene. Plasmid pFAJ1819 can be replicated in *E. coli* S17 λ-pir but not in *D. solani* cells (Xi et al., 1999). *D. solani* strain IPO2222 (van der Wolf et al., 2014) was grown at 28°C on tryptic soya agar (TSA; Oxoid), in tryptic soya broth (TSB; Oxoid) or in M9 minimal medium (MP Biomedicals) supplemented with glucose (Sigma-Aldrich) to a final concentration of 0.4%. To solidify the media, 15 g L^−1^ bacteriological agar (Oxoid) was added. When required, the growth media were further supplemented with neomycin (Sigma-Aldrich) to a final concentration of 50 μg ml^−1^ and with X-gluc (5-bromo-4-chloro-3-indolyl-β-D-glucuronide; GeneON) to a final concentration of 20–100 μg ml^−1^. *In vitro* plants of *S. tuberosum* cv. Kondor were obtained from the Laboratory of Seed Production and Potato Protection, Plant Breeding, and Acclimatization Institute – National Research Institute, Bonin, Poland and *in vitro* plants of *S. dulcamara* (accession: A54750008, The Experimental Garden and Genebank, Radboud University Nijmegen, the Netherlands) were obtained from Prof. Titti Mariani from Department of Molecular Plant Physiology, Radboud University, Nijmegen, the Netherlands. Plants were grown on Murashige and Skoog (MS) medium (Murashige and Skoog, 1962) supplemented with Gamborg’s vitamin mixture (Duchefa Biochemie bv.) (MS+G vit.), 30 g L^-1^ sucrose (Chempur), pH 5.8, and solidified with 7 g L^−1^ plant agar (Duchefa Biochemie bv.) at 22°C under 16/8-h (day/night) photoperiod as previously described (Fikowicz-Krosko and Czajkowski, 2017b).

**Table 1:** Genetic loci of *D. solani* strain IPO2222 Tn5 mutants regulated in the presence of *S. tuberosum* and *S. dulcamara* tissues.

No.	Mutant	Insertion name, Tn5 locus	Protein accession number, protein name	Transcriptional organization (single gene vs. operon) ^A^	Entry, KEGG pathway, UniProt-based protein function	Average fold induction in contact with (± SD):						
						*S. tuberosum:*				*S. dulcamara:*		
						leaf	stem	roots	tuber	leaf	stem	roots
**1**	M32	***ds32***, *garD*	WP_099048964.1, galactarate dehydratase	single gene	EC: 4.2.1.42, ascorbate and aldarate metabolism	**5.1** (± 0.3)	**1.9** (± 0.3)	0.2 (± 0.4)	0.5 (± 0.6)	**2.4** (± 0.2)	0.2 (± 0.2)	0.1 (± 0.0)
**2**	M83	***ds83***, *fcl*	WP_022634990.1, GDP-L-fucose synthase, colanic acid biosynthesis protein WcaG	operon: contains 7 genes (*cpsB*, *cpsG*, *gmd*, *fcl*, *wbeA*, *rfbD*, *and wcaG*)	EC:1.1.1.271, fructose and mannose metabolism, amino sugar and nucleotide sugar metabolism	**4.7** (± 0.4)	**1.6** (± 0.1)	0.2 (± 0.3)	**14.3** (± 1.1)	**8.3** (± 0.7)	**4.2** (± 0.4)	**3.5** (± 0.3)
**3**	M363	***ds363***, *nrdD*	WP_022631886.1, anaerobic ribonucleoside-triphosphate reductase	operon: contains 2 genes (*nrdD* and *nrdG*)	EC:1.1.98.6, purine and pyrimidine metabolism	0.1 (± 0.1)	0.2 (± 0.3)	0.7 (± 0.7)	**7.5** (± 0.5)	0.1 (± 0.2)	0.6 (± 0.4)	**4.6** (± 0.1)
**4**	M481	***ds481***, A4U42_RS17300	WP_080644163.1, ATP-binding protein/histidine kinase	data not available	entry no assigned, UniProt not assigned	0.2 (± 0.1)	**4.1** (± 0.5)	0.6 (± 0.5)	**15.1** (± 0.5)	**11.3** (± 0.8)	**10.6** (± 0.4)	0.5 (± 0.1)
**5**	M605	***ds605***, A4U42_RS15880	WP_022632810.1, DUF4123 domain-containing protein	data not available	entry no assigned, UniProt not assigned	0.1 (± 0.2)	**5.1** (± 0.4)	0.5 (± 0.5)	0.3 (± 0.3)	0.2 (± 0.2)	0.2 (± 0.4)	0.3 (± 0.3)
**6**	M691	***ds691***, *ganL*	WP_022634306.1, maltoporin	single gene	entry no assigned, maltose transporting porin activity, maltodextrin transmembrane transporter activity	**2.9** (± 0.4)	0.4 (± 0.3)	**3.4** (± 0.4)	**3.6** (± 0.5)	**8.8** (± 0.2)	**8.2** (± 0.6)	**2.5** (± 0.3)
**7**	M713	***ds713***, *gmd*	WP_022634987.1, GDP-mannose 4,6-dehydratase	operon: contains 7 genes (*cpsB*, *cpsG*, *gmd*, *fcl*, *wbeA*, *rfbD*, *and wcaG*)	EC:4.2.1.47, fructose, mannose, amino sugar and nucleotide sugar metabolism	0.2 (± 0.2)	0.2 (± 0.3)	0.0 (± 0.1)	**3.4** (± 0.3)	0.2 (± 0.3)	**2.2** (± 0.3)	**1.6** (± 0.1)
**8**	M741	***ds741***, *thiJ*	WP_022633614.1, type 1 glutamine amidotransferase domain-containing protein	single gene	entry no assigned, ThiJ/PfpI family protein, integral component of membrane	**2.6** (± 0.3)	0.3 (± 0.3)	**2.8** (± 0.7)	**3.0** (± 0.3)	0.4 (± 0.3)	0.1 (± 0.1)	0.2 (± 0.3)
**9**	M743	***ds743***, A4U42_RS19150	WP_022633459.1, hypothetical protein	data not available	entry no assigned, uncharacterized protein	0.1 (± 0.1)	0.1 (± 0.1)	0.3 (± 0.3)	0.1 (± 0.2)	**4.6** (± 0.3)	**4.3** (± 0.1)	**7.1** (± 0.4)
**10**	M748	***ds748***, *pstB*	WP_022635502.1, phosphate ABC transporter ATP-binding protein PstB	operon: contains 3 genes (*pstB*, *pstA*, and *pstC*)	EC:7.3.2.1, ABC transporter, phosphate and amino acid transporters, part of the ABC transporter complex PstSACB involved in phosphate import, responsible for energy coupling to the transport system.	0.5 (± 0.3)	0.2 (± 0.3)	**2.6** (± 0.4)	**3.3** (± 0.2)	**7.0** (± 0.2)	**7.5** (± 0.3)	**6.9** (± 0.2)
**11**	M754	***ds754***, A4U42_RS04765	WP_026594537, HAMP domain-containing protein, putative chemotaxis receptor	single gene	entry no assigned, methyl-accepting chemotaxis protein I, serine chemoreceptor protein	0.4 (± 0.5)	0.3 (± 0.3)	0.1 (± 0.3)	0.4 (± 0.3)	**5.6** (± 0.3)	**6.8** (± 0.2)	**9.9** (± 0.3)
**12**	M814	***ds814***, *kdgN*	WP_022633504.1, KdgN; oligogalacturonate-specific porin protein	single gene	entry no assigned, N-acetylneuraminic acid outer membrane channel protein NanC	0.7 (± 0.5)	0.5 (± 0.6)	0.6 (± 0.6)	**8.1** (± 0.7)	**7.7** (± 0.3)	**9.6** (± 0.5)	**8.0** (± 0.3)
**13**	M1032	***ds1032***, A4U42_RS05240	WP_023637989.1, glucosamine kinase	operon: contains 3 genes (2 copies of *gpsK* gene and a nameless gene coding for N-acetyl -D-glucosamine ABC transporter sugar-binding protein)	entry no assigned, glucosamine kinase GpsK	0.2 (± 0.3)	0.3 (± 0.4)	0.7 (± 0.5)	**4.9** (± 0.3)	**5.6** (± 0.4)	**6.8** (± 0.5)	0.2 (± 0.2)

Induction measured as the average change of the relative GUS expression under inductive to non-inductive conditions. The GUS activity was assessed by qualitative spectrophotometric analyses of p-nitrophenyl-β-D-glucuronide enzymatic degradation by GUS and the release of p-nitrophenol. Each mutant was tested in four independent experiments (four replicates, n = 4) per treatment and the results were averaged. Relative fold ≥ 1.5 is marked in bold. SD, standard deviation calculated from four replicates.

^A^Assessment of the transcriptional organization was predicted using DOOR: a database of prokaryotic operons accessed via http://161.117.81.224/DOOR3/index.php (Dam et al., 2007; Mao et al., 2009). The complete genome of D. dadantii strain 3937 (Genbank accession NC_014500) (Glasner et al., 2011) was used as a reference.

### Transposon Mutagenesis With mini-Tn5

Random transposon mutagenesis *via* conjugation of *D. solani* strain IPO2222 with *E. coli* strain S17 λ-pir pFAJ1819-mini-Tn5 was done as described previously (Czajkowski et al., 2017). The Tn5 transfer rate (conjugation rate of the pFAJ1819 plasmid carrying the mini-Tn5 transposon from *E. coli* to *D. solani* cells) was determined using the equation: X = (Ir × 100)/Id; where X is the conjugation rate, Ir is the density of the recombinants (Tn5 mutants) in the conjugation mixture (cfu/ml), and Id is the density of the donors (cfu/ml) in the conjugation mixture. The experiment was independently repeated three times and the results were averaged. The Clark-Carbon equation [P = 1 − (1 − f)^N] (Pérez-Ortín et al., 1997), [where *P* is the probability to find a gene with desired function (in this study, a gene containing a Tn5 transposon), *f* is a fraction of the genome (if the average gene in *D. solani* is 1,200-bp long and the *D. solani* IPO2222 complete genome is 4,919,833 bp. (Khayi et al., 2016) – f = 1,200/4,919,833 = 0.00024391) and N is the number of the tested IPO2222 Tn5 mutants (in this study N = 10,000)], was used to determine the coverage of the IPO2222 genome with Tn5 transposition events in the mutagenesis assays as previously described (Lisicka et al., 2018).

### Southern Hybridization Analyses of the Tn5 Insertions

To determine the number of Tn5 insertions per *D. solani* genome, Southern hybridization analysis was performed as described earlier (Sambrook et al., 1989; Czajkowski et al., 2017). In brief, genomic DNA from selected *D. solani* Tn5 mutants was isolated using a Wizard Genomic DNA Purification Kit (Promega) using the protocol provided by the manufacturer. Southern blot transfer of bacterial genomic DNA digested with *Pst*I restriction endonuclease was performed according to Sambrook et al. (1989). A 679-bp PCR product of the *gusA* gene was used as the hybridization probe as described earlier (Czajkowski et al., 2017) and the hybridization and detection were performed according to the protocol of the digoxigenin DNA-labeling and detection kit (Roche Diagnostics GmbH).

### Verification of the *D. solani* Tn5 Mutants by *Dickeya* spp. Specific-PCR and Plating on CVP Medium

PCR-based identification of selected *D. solani* Tn5 mutants was performed using *Dickeya* spp.–specific primers Df (5’- AGAGTCAAAAGCGTCTTG-3’) and Dr (5’-TTTCACCCACCGTCA GTC-3’) (Laurila et al., 2008). Bacterial genomic DNA for PCR was prepared as previously described (Czajkowski et al., 2010b). Amplified DNA fragments were detected by electrophoresis on a 0.5 × TBE 1.5% agarose gel stained with 50 μg ml^−1^ GelRed (Biotium). The ability of *D. solani* Tn5 mutants to form cavities (pits) on crystal violet pectate medium (CVP) was tested as described elsewhere (Hélias et al., 2011).

### Glucuronidase Activity of *D. solani* Tn5 Mutants Under Noninductive Conditions

Beta-glucuronidase (GUS) activity of the Tn5 bacterial mutants was initially assessed visually 48 h after inoculation by the intensity of blue coloration of bacterial colonies growing at 28°C on M9 agar plates supplemented with neomycin to a final concentration of 50 μg ml^−1^and X-gluc to a final concentration of 20 μg ml^−1^. The Tn5 mutants exhibiting a GUS positive phenotype (blue color of the colonies) on M9 plates under non-inductive conditions (absence of plant tissues) were removed from further analyses (Czajkowski et al., 2017).

### Propagation of *S. tuberosum* and *S. dulcamara In Vitro* Plants and Qualitative Screening for Plant Tissue-Induced Gene Expression of *D. solani* Tn5 Mutants

*In vitro* growth and propagation of the *S. tuberosum* and *S. dulcamara* plants were done as previously described (Czajkowski et al., 2015; Fikowicz-Krosko and Czajkowski, 2017b). Plants were incubated under growth chamber conditions in SMC-250-CC phytochamber (Sanwood Technologies, China) equipped with photosynthetic light banks (Sanwood D65, 15W, 8500K) under 16/8-h light/dark regime and at temperature of 22 ± 0.5°C. The *D. solani* Tn5 mutants were screened for plant tissue–specific gene expression using a previously established protocol (Fikowicz-Krosko and Czajkowski, 2017a). Each Tn5 mutant was tested for the GUS positive phenotype under seven experimental conditions: in the presence of leaves, stems, roots, and mini-tubers of *S. tuberosum* (four conditions) as well as in the presence of leaves, stems and roots of *S. dulcamara* (three conditions). As a negative control, *D. solani* Tn5 mutants growing in the absence of plant tissues as well as the wild-type strain IPO2222 not expressing GUS activity were used.

### Quantitative Assessment of Gene Expression in *D. solani* Tn5 Mutants by Measuring GUS Activity

GUS activity was quantified after overnight incubation (ca. 12 h) by a spectrophotometric assay using p-nitrophenyl-*β*-D-glucuronide (Sigma-Aldrich) as a substrate as described elsewhere (Wilson et al., 1995; Czajkowski et al., 2017). Total protein content was determined using a Pierce BCA protein assay kit (ThermoFisher Scientific) according to a protocol provided by the manufacturer. GUS activity was expressed as pmol of the product (p-nitrophenol) per min per mg of total protein. Tn5 mutants showing a statistically significant increase of GUS activity in the presence of plant tissues—at least 1.5-fold difference (150%) in comparison with GUS activity under noninductive conditions, as advised earlier (Czajkowski et al., 2017; Fikowicz-Krosko and Czajkowski, 2017a), were retested under the same conditions with four replicates per isolate and treatment, and selected for further experiments.

### Identification of the Tn5 Insertion Sites by Genome Sequencing and Analysis of the Function of the Disrupted Genes

In order to in detail localize the Tn5 insertion sites in the genomes of *D. solani* mutants, the genomes of selected mutants were sequenced. For this, the bacterial genomic DNA was obtained with the use of a Wizard Genomic DNA Purification Kit (Promega) using the protocol provided by the manufacturer. The genomic DNA of each mutant was sequenced and assembled into draft genome at the Laboratory of DNA Sequencing and Oligonucleotide Synthesis at the Institute of Biochemistry and Biophysics of the Polish Academy of Science, Warsaw, Poland using Illumina technology. Structural and functional annotations of draft genomes were obtained from RAST (Rapid Annotation using Subsystem Technology (http://rast.nmpdr.org/) (Aziz et al., 2008). The location of the Tn5 insertions in the draft genomes of *D. solani* IPO2222 Tn5 mutants was determined from BlastN and BlastX alignments (http://blast.ncbi.nlm.nih.gov/Blast.cgi) (Altschul et al., 1990). Using the available complete genome sequence of *D. solani* strain IPO2222 (Khayi et al., 2016) and the draft genomes of the Tn5 mutants, the localization of the Tn5 transposon in the bacterial chromosome was in detail assessed. For each mutant, at least ca. 1,000- to 5,000-bp-long sequences flanking the Tn5 insertion were analyzed to determine the genomic context of the each of Tn5-distruped gene (Lisicka et al., 2018). The putative function of the disrupted genes was inferred using BlastN and BlastX alignments accessed as described above. Furthermore, the functions of the unknown genes (open reading frames encoding hypothetical proteins or proteins with no homology to known proteins) were analyzed using GeneSilico Protein Structure Prediction meta-server, containing known three-dimensional (3D) protein structures (Kurowski and Bujnicki, 2003), together with PSI-BLAST (Altschul and Koonin, 1998) accessed from NCBI. The predicted functions with the highest scores were considered as the most probable.

### Quantification of the Expression of Selected *D. solani* Genes Using qRT-PCR

Expression of *D. solani* IPO2222 genes, identified in Tn5 mutants expressing a plant-dependent GUS phenotype, as well as the two reference genes *lpxC* and *yafS* (Hommais et al., 2011) was analyzed under seven experimental conditions using qRT-PCR. For this, *D. solani* IPO2222 overnight cultures in M9 medium supplemented with 0.4% glucose were incubated for ca. 16 h at 28°C with shaking (200 rpm) and cultures containing ca. 10^9^ cfu ml^−1^ were diluted 1:50 (v/v) in the same fresh medium. Twenty millimeters of the diluted bacterial culture was aseptically transferred to a sterile 50-ml Falcon tube (Sarstedt) into which 20 leaves, eight leafless 8- to 10-cm-long stem fragments, 3 g of plant roots or 4 potato mini-tubers taken from the *in vitro* cultivated plants were individually added. The tubes were then incubated for 16 h at 28°C with gently shaking (50 rpm). After this time, the plant material was discarded, and 10 ml of bacterial culture was individually transferred to a 15-ml Falcon tube (Sarstedt). OD measurements (λ= 600 nm) were used to estimate bacterial abundance for RNA isolation (ca. 10^9^ cells). Bacterial cells were pelleted by centrifugation (8,000 × g, 2 min.) and per sample, the pellet was suspended in 500 μl of the RNA stabilizing agent (Amplicon, Poland) according to protocol provided by the manufacturer (Amplicon, Poland). Five biological replicates were prepared per each experimental condition to be tested. The negative control was five biological replicates of the *D. solani* cultures grown for 16 h under the same conditions but without supplementation with plant tissue fragments and normalized for cell abundance in a similar manner. Primer design, RNA isolation, DNA removal, RNA quantification and normalization as well as statistical analyses of the gene expression patterns were obtained commercially from Amplicon, Poland (https://en.amplicon.pl/). Briefly, total RNA was isolated with a GeneMATRIX Universal RNA Purification Kit (EURx) according to protocols provided by the manufacturer. Residual DNA was removed with DNase I (6U/sample; EURx). RNA quality and purity were assessed by NanoDrop spectrophotometer (Thermo Scientific) and agarose gel electrophoresis. cDNA was obtained from the isolated total RNA with the use of a NG dART RT kit (EURx) and protocol provided by the manufacturer. Reverse transcription was done in the total volume of 20 μl using 800 ng of RNA per sample and qPCR was done with the use of SG qPCR Master Mix (EURx) with the protocol provided by the manufacturer in the thermal cycler LightCycler 480 II (Roche). Primer sequences are shown in **Supplementary Table 3**. The relative, average expression levels of the candidate genes in the five biological replicates per treatment were calculated using ΔΔCt method (Livak and Schmittgen, 2001). The expression levels of the candidate genes were normalized with the use of the expression levels of the two reference genes *lpxC* and *yafS* (Hommais et al., 2011). To statistically analyze the qRT-PCR data the following statistical tests were done: the Dixon’s Q test (p < 0.05) (Dean and Dixon, 1951) was used for identification and rejection of outliers. The levels of expression for treatment transcripts were scaled in order to obtain an average level of expression in the reference group equal to 1 (100%). The Shapiro-Wilk test (p < 0.05) (Shapiro and Wilk, 1965) was applied to test normality of results distribution for individual transcripts. The homogeneity of variance was verified using the Fisher-Snedecor test (Box, 1953) or the Ansari-Bradley (Lepage, 1971) test, if normal distribution was not followed. The mean expression levels for each treatment sample and the appropriate reference sample were compared using the two-tailed Student’s t-test (p < 0.05) (Student, 1908) when both groups were normally distributed. Otherwise, the Mann Whitney-Wilcoxon test (Wilcoxon, 1945) was used.

### Transcriptional Organization, Biochemical Pathways, and Cellular Enzymatic Networks Affected by the *D. solani* Tn5 Insertions

The transcriptional organization (transcript of the individual gene *vs.* transcript as a part of the operon) was predicted for 13 *D. solani* IPO2222 genes containing Tn5 insertions. These predictions have been done with the use of DOOR: a database of prokaryotic operons accessed *via* http://161.117.81.224/DOOR3/index.php (Dam et al., 2007; Mao et al., 2009). The complete genome sequence of *D. dadantii* strain 3937 (Genbank accession NC_014500) (Glasner et al., 2011) was used as a reference. Analyses of the biochemical pathways in which the selected genes might participate were done using KEGG (Kanehisa and Goto, 2000). The results were visualized using iPath (Letunic et al., 2008). Similarly, proteins were evaluated for their predicted biological, functional, and metabolic roles in cellular networks using STRING (Search Tool for Retrieval of Interacting Genes/Proteins) v11 (https://string-db.org/) providing essential information regarding interactions of proteins of interest (Szklarczyk et al., 2019) using the proteome of *D. dadantii* strain 3937 as a reference.

### Assessment of Cell and Colony Morphology of Selected *D. solani* Mutants With Microscopic Techniques

The morphology of bacterial cells was assessed using transmission electron microscopy (TEM) as previously described (Czajkowski et al., 2017). TEM analysis was performed by the Laboratory of Electron Microscopy, Faculty of Biology, University of Gdansk, Poland. Likewise, the morphology of bacterial colonies of selected *D. solani* Tn5 mutants was analyzed using a Leica MZ10F stereomicroscope at 10× and 40× magnifications coupled with a Leica DFC450C camera system (Leica) as previously described (Lisicka et al., 2018).

### Phenotypic Characterization of *D. solani* Tn5 Mutants Using BIOLOG Phenotypic Microarrays

Selected *D. solani* IPO2222 mutants were analyzed using the BIOLOG phenotypic microarray system, with GEN III and EcoPlate microplates (Biolog Inc.). Each GEN III plate contains 94 phenotypic tests, i.e., 71 carbon source utilization assays and 23 chemical sensitivity assays. Each EcoPlate contains 31 different complex carbon sources (www.biolog.com). Bacterial cultures were grown on TSA for 24 h at 28°C and resuspended into inoculation fluid (IF-A) (GENIII) or into 10 mM phosphate buffer pH 7.0 (EcoPlate) using a sterile cotton swab. Turbidity of the suspension was adjusted to ca. 90% T with the use of a spectrophotometer [A = log(%T)]. One hundred microliters of suspensions were inoculated into each well of the microplates using a multichannel pipette. Inoculated plates were sealed with parafilm and incubated for 24 h at 28°C. The wells were then observed for a color change (positive reaction). Color development was also recorded using an Epoch2 microplate spectrophotometer (BioTek) using a λ = 570-nm wavelength filter. Plates inoculated with the wild-type *D. solani* strain IPO2222 were used as controls.

### Phenotypic Characterization of *D. solani* Tn5 Mutants Using Plate Assays

Selected *D. solani* Tn5 mutants were screened for various phenotypic features, putatively important for colonization and infection of plant tissues. These included chemotaxis (de Weert et al., 2002), biofilm formation (Nykyri et al., 2013), production of quorum sensing signal molecules (Latifi et al., 1995; Czajkowski et al., 2012), cell generation time in rich and minimal media (Roth, 2006), growth in the presence of plant extracts and under anaerobic conditions (Czajkowski et al., 2012) and for ability to grow with mannose, maltose or galactaric acid as a sole carbon source (Lisicka et al., 2018). The ability to cause rotting of chicory leaves and potato tubers was evaluated as described in (Czajkowski et al., 2012; Krzyzanowska et al., 2012). The detailed experimental protocols of these tests are provided as Supplementary Material.

### Analyses of *S. tuberosum* and *S. dulcamara* Extracts by Mass Spectrometry

Mass spectrometry analyses of *S. tuberosum* and *S. dulcamara* extracts were done to assess the presence and concentration of known antibacterial peptides and other plant products (carbon and nitrogen sources) (Parachin and Franco, 2014; Bártová et al., 2019) that could influence the interaction including chemotaxis and bacterial growth in the presence of plant extracts of *D. solani* wild-type and Tn5 mutants with host plants. The detailed experimental protocol of these analyses is provided in Supplementary Materials.

### Host Colonization and Virulence of Selected *D. solani* Tn5 Mutants on *S. tuberosum* and *S. dulcamara* Plants Grown in Culture Tubes

Selected *D. solani* Tn5 mutants were assessed for the ability to colonize and to cause symptoms on tissue-cultured *S. tuberosum* and *S. dulcamara* plants. Plants were cultivated and propagated as described above. Briefly, for the host colonization and virulence experiments, the apical nodes of *S. tuberosum* and *S. dulcamara* plants with at least two pair of leaves per plant were harvest from ca. 1 month-old plant culture stocks and placed on the new plant growth medium in individual culture tubes. After 14 days, when plants were ca. 4- to 6-cm high, with developed at least 1-cm-long roots and with at least 4 to 6 pairs of leaves per plant, they were selected for inoculation (Fikowicz-Krosko and Czajkowski, 2017b). *In vitro* plants (total n = 40, 20 plants per experiment and per plant species replicated one time) were infected with *D. solani* Tn5 mutants by inoculation of bacterial cultures onto base of each plant just above the MS medium as described earlier (Czajkowski et al., 2015). Each mutant was tested in duplicate on 10 *S. tuberosum* and 10 *S. dulcamara* plants grown in individual culture tubes De Wit (Duchefa Biochemie bv.). The experiment was repeated once. Inoculated plants were visually inspected for symptom development at 14 days post inoculation (dpi) as described earlier (Fikowicz-Krosko and Czajkowski, 2017b).

### Statistical Analyses

Bacterial counts were analyzed with ordinary linear regression using the statistical software package Past3 (Hammer et al., 2001). To achieve normality data were transformed as log_10_ (x + 1). Results were considered to be significant at p ≤ 0.05 and pair-wise differences were obtained using the *t*-test. For experiments involving the *in vitro* plants, data were analyzed according to the experimental design in which two replicated experiments were done per each treatment of replicated plants. The adopted linear model was a complete block design with replicates as blocks, and the main effects analyzed for the contribution of time and treatment and the two-way interaction between time and treatment. A normal distribution of plant height and weight was assumed.

## Results

### Transposon Mutagenesis and Identification of Genes/Operons Expressed Exclusively in the Presence of *S. tuberosum* and/or *S. dulcamara* Tissues

**Figure 1:**
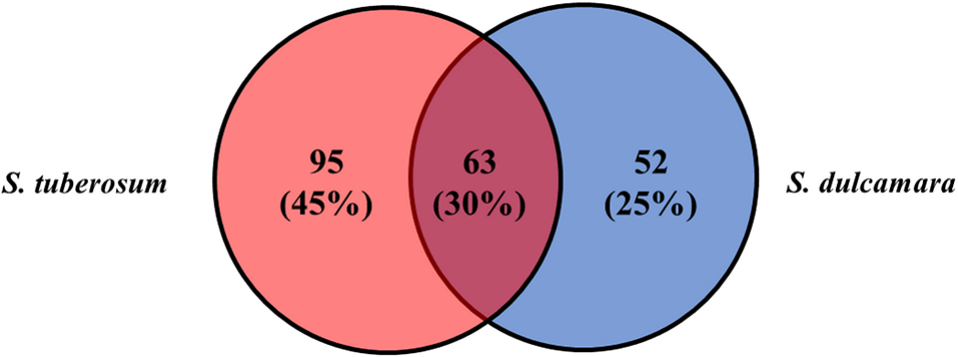
*D. solani* IPO2222 Tn5 mutants in genes up-regulated in the presence of crop host—*S. tuberosum* (pale red) and alternative, weed host—*S. dulcamara* (pale blue). From a pool of 10,000 mutants screened, 210 Tn5 mutants demonstrated elevated GUS expression in the presence of either *S. tuberosum* and *S. dulcamara* or both plant species as it was qualitatively assessed as a development of blue color resulted from cleavage of X-gluc (glucuronidase substrate) by GUS enzyme. Sixty-three IPO2222 Tn5 mutants were up-regulated by the presence of plant species derived from both plants screened (purple).

**Figure 2:**
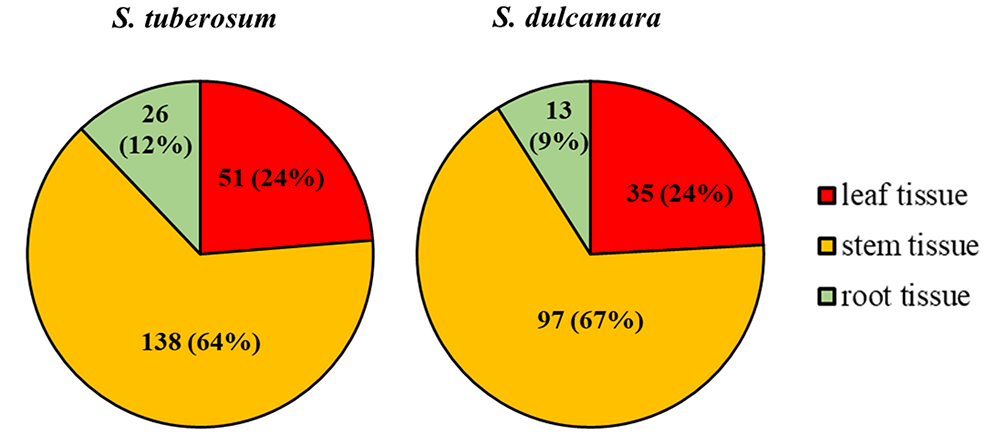
*D. solani* IPO2222 Tn5 mutants in genes up-regulated in the presence of *S. tuberosum* and/or *S. dulcamara* plant tissues. The 210 confirmed Tn5 bacterial mutants that expressed a GUS-positive phenotype in the presence of at least one type of plant tissue (leaf, stem and/or root tissue) derived from *S. tuberosum* and *S. dulcamara* plants as it was qualitatively assessed as a development of blue color resulted from cleavage of X-gluc (glucuronidase substrate) by GUS enzyme. The total number of mutants does not add up to 210 as some of the mutants were up-regulated by the presence of more than one plant tissue tested.

A total of 10,000 Tn5 transposon mutants of *D. solani* strain IPO2222 were generated and screened for differential expression of a GUS reporter gene in bacterial cells both in the presence and absence of *S. tuberosum* and *S. dulcamara* tissues. Assuming a random distribution of insertions, approximately 90% of the genes present in the IPO2222 genome contained at least one insertion of the Tn5-*gusA* transposon (Lisicka et al., 2018). GUS activity for all mutants was initially assessed visually under non-inductive conditions on M9 + glucose minimal medium to identify those mutants expressing a reporter gene in the absence of plant tissues. A total of 969 mutants (9.7%) did not express GUS activity in the absence of plants and these were retained for further analysis. Of these, 210 exhibited a GUS positive phenotype in the presence of one or another or both plant species (**Figure 1**) as well as in the presence of leaf, stem, root, or tuber tissue of either *S. tuberosum* or *S. dulcamara* tissues or both (**Figure 2**). Quantitative GUS assays of these 210 mutants revealed that the 13 Tn5 mutants exhibited the highest increase in relative GUS activity upon exposure to one of the plant environments. The genomes of these 13 mutants were sequenced (the draft genome sequences are available as Supplementary data) to identify the transposon insertion sites. A single insertion of the Tn5 transposon per genome was observed for each of the 13 mutants as confirmed by Southern blot analysis (data not shown) and genome sequencing.

### Up-Regulation of *D. solani* Genes/Operons in the Presence of *S. tuberosum* and/or *S. dulcamara* Plant Tissues Analyzed With qRT-PCR

**Figure 3:**
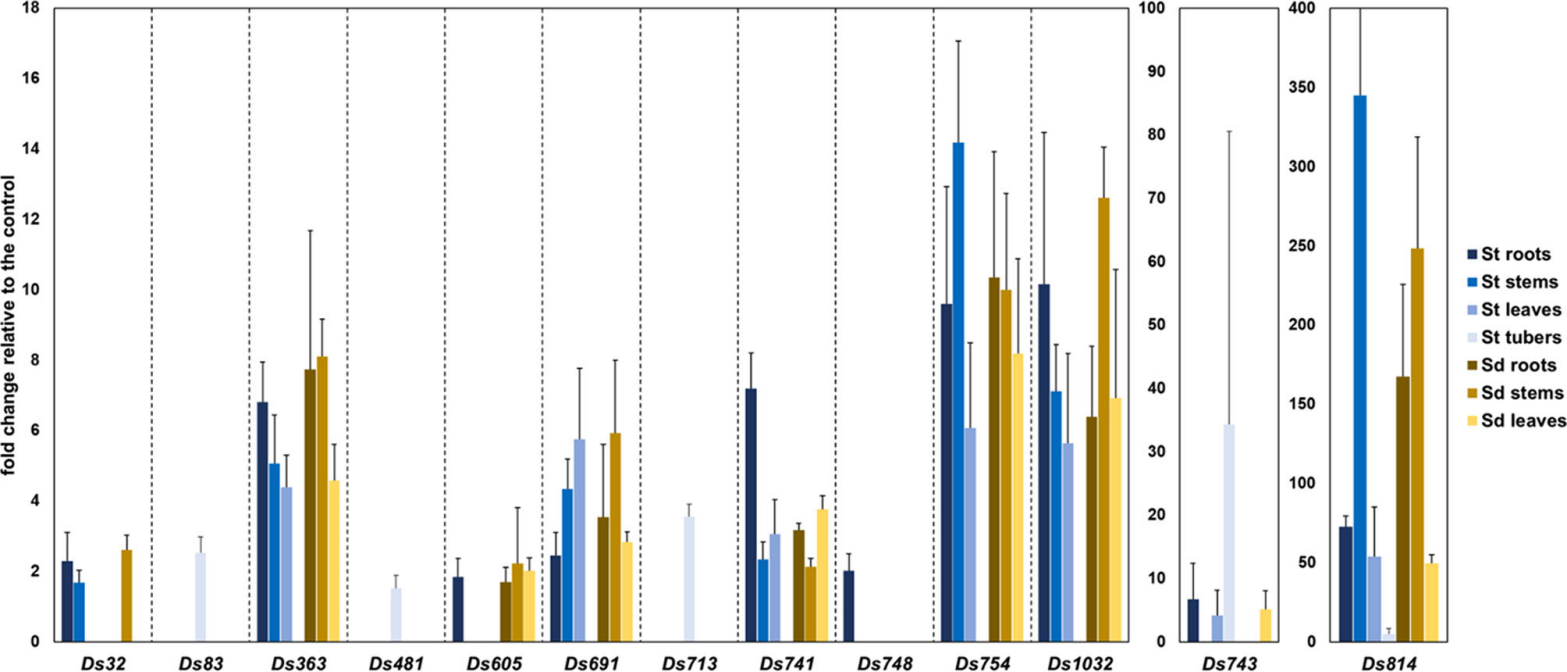
qRT-PCR analysis of the 13 *D. solani* loci found to be up-regulated in the presence of *S. tuberosum* and/or *S. dulcamara* plant tissues. Per locus, the fold change under inductive to non-inductive conditions normalized to the expression of the control genes is shown. Five biological replicates were analyzed per locus and the results were averaged. Expression of each gene was analyzed under seven conditions. St, *Solanum tuberosum*; Sd, *Solanum dulcamara*.

All 13 candidate mutants having apparent elevated expression in qualitative GUS assays in the presence of tissue of *S. tuberosum* or *S. dulcamara* tissues (**Table 1**) exhibited plant tissue-regulated expression when assessed by qRT-PCR (**Figure 3**). The relative gene/operon induction measured by qRT-PCR ranged from 1.5- to 345-fold, depending on the particular transcriptional unit examined and/or the plant tissue to which bacterial cells were exposed (**Figure 3**). Substantial variation in the plant species and tissue-dependent induction was observed among these 13 target units. Only one of these units (insertion *ds814*) was up-regulated in the presence of all tissue types of both plant species examined. Five others (insertions *ds363*, *ds691*, *ds741*, *ds754*, and *ds1032*) were induced in the presence of all *S. tuberosum* and *S. dulcamara* tissues with the exception of potato tubers. In contrast, three transcriptional units (insertions *ds83*, *ds481*, and *ds713*) were up-regulated only in the presence of *S. tuberosum* tuber tissue but not in the presence of any *S. dulcamara* tissues screened. Insertion *ds748* was up-regulated only in the presence of *S. tuberosum* roots while three other insertions (*ds32*, *ds605*, and *ds743*) were induced by three to four types of tissues among the seven tested of these two plant species (**Figure 3**).

### Characterization of the Tn5 Insertion Sites in *D. solani* Mutants

The DNA sequences flanking the inserted Tn5 transposons of the 13 mutants (ca. 1,000 to 5,000 bp) were analyzed using BlastP to acquire further insight of the genetic context of the genes disrupted by Tn5 insertion. These plant-inducible loci were found to encode membrane proteins, proteins involved in primary metabolism and/or chemotaxis, as well as some hypothetical proteins (**Table 1**, **Supplementary Table 1**, **Supplementary Table 2**, and **Supplementary Figure 1**).

From the 13 *D. solani* IPO2222 transcriptional units, interrogated for their transcriptional organization, 5 (insertions *d32*, *d691*, *d741*, *d754*, and *d814*) were predicted to be transcribed in a form of an individual gene, whereas 5 other (insertions *d83*, *d363*, *d713*, *d748*, and *d1032*) were predicted to be parts of the operons. Interestingly, two such units (insertions *d83* and *d713*) were predicted to be a part of the same operon. The transcriptional organization of three units (insertions: *d481*, *d605*, and *d743*) could not be assessed using the DOOR operon database (**Table 1**). In the qRT-PCR, the insertions *d83* and *d713* expressed the same gene induction pattern—both genes were induced exclusively in the presence of potato tuber tissue as well as were not induced in the presence of all other plant tissues tested. Likewise, the M83 and M713 mutants expressed similar phenotypes (**Supplementary Table 2**).

Examination of the KEGG biochemical pathways corresponding to these 13 transcriptional units enabled assignment of some to the cellular pathways involved in: (i) organic acid and sugar metabolism (insertions *ds32*, *ds83*, *ds691*, and *ds713*), (ii) purine and pyrimidine metabolism (insertion *ds363*), (iii) chemotaxis (insertion *ds745*), (iv) phosphorylation (insertion *ds481*), and (v) transport across the outer membrane (insertion *ds748* and *ds814*) (**Supplementary Table 1** and **Supplementary Figure 1**).

Likewise, the putative interacting partners of the 13 *D. solani* selected proteins were assessed using STRING (**Supplementary Table 1**). Although, the transcriptional units induced by *S. tuberosum* and/or *S. dulcamara* were randomly located in the genome of *D. solani* IPO2222 (**Table 1**), they were often found in a close proximity to genes/operons encoding proteins likely involved in plant-microbe interactions, *viz*., stress response, chemotaxis and sugar metabolism and acquisition (**Supplementary Table 1**).

### Phenotypes of *D. solani* Tn5 Mutants

The 13 Tn5 mutants exhibiting significant differential response to the presence of plant tissues were screened in plate assays for phenotypes distinct from the phenotype of wild-type *D. solani* strain IPO2222. Results from all phenotypic tests are summarized in **Supplementary Table 2**.

Briefly, no differences were observed among the 13 mutants and wild-type IPO2222 strain in the majority of metabolic phenotypes examined with the use of BIOLOG GENIII and Ecoplate microarrays. The collection of 13 mutants differed from the wild-type strain in a total of only 8 features of 94 tested with Biolog GEN III plates and in 2 features from 31 tested using Biolog EcoPlates. All 13 mutants lost their ability to utilize glucose-1-phosphate. Furthermore, nine mutants [all except M481 (insertion: *ds481*), M691 (*ds691*), M741 (*ds741*), and M814 (*ds814*)] acquired the ability to use D-sorbitol and nine mutants [all except M83 (*ds83*), M363 (*ds363*), M713 (*ds713*), and M754 (*ds754*)] developed the ability to use l-alanine as a carbon source, respectively. In addition, mutant M83 showed susceptibility to formic acid and 4% NaCl and mutant M32 was unable to assimilate galactaric acid and developed susceptibility to 4% NaCl. Likewise, due to the Tn5 insertion, mutant M713 gained the ability to use D-turanose, α-D-lactose, N-acetyl-β-D-mannosamine and D-sorbitol as carbon sources but developed susceptibility to 4% NaCl. *D. solani* strain IPO2222 was able to grow with mannose or galactaric acid but not with maltose as a sole carbon source. Most Tn5 mutants maintained these phenotypes; they were able to use mannose and galactaric acid and were unable to use maltose to grow. All mutants except M32 kept the ability to use galactaric acid as a sole carbon source. Likewise, all mutants sustained the ability to form biofilm in similar abundance and maintained the ability to produce similar amounts of AHLs as the wild-type strain. In addition, the mutants all grew equally well as IPO2222 wild-type strain under anaerobic conditions.

Furthermore, no visible differences in cell morphology were seen in the mutants in comparison with the wild-type strain IPO2222 using transmission electron microscopy except that of mutant M691 which had elongated cells. All colonies of the Tn5 mutants exhibited colony morphology and diameter similar to those of the *D. solani* wild-type strain IPO2222 (= ca. round colonies of 0.3 to 0.6 cm in diameter with undulate margins, crateriform and milky in color).

The average generation time of *D. solani* IPO2222 in TSB (rich medium) and M9 + glucose (minimal medium) under our experimental conditions was ca. 102 min (± 5%) (1:42 h) and ca. 134 min (± 5%) (2:14 h), respectively. In comparison with the wild-type strain IPO2222, none of the tested mutants expressed statistically significant growth change in both tested media. Furthermore, all 13 selected Tn5 mutants displayed similar growth as the wild-type strain in M9+glucose minimal medium supplemented with *S. tuberosum* or *S. dulcamara* plant extracts.

Seven mutants (M32, M83, M605, M713, M741, M814, and M1032) showed a similar chemotactic attraction as the wild-type strain to extracts from both plant species. Two mutants, M481 and M754, showed no chemotactic response toward either plant extract. Three other mutants (M363, M743, and M748), exhibited no chemotaxis toward the *S. dulcamara* extract but were attracted by the extract of *S. tuberosum*.

### Proteomic Analysis of the *S. tuberosum* and *S. dulcamara* Extracts

Mass spectrometry analyses of the *S. tuberosum* and *S. dulcamara* plant extracts allowed identification of 138 and 200 unique plant proteins in the extracts, respectively. The mass spectrometry proteomics data were deposited to the Proteome Xchange Consortium *via* the PRIDE (Perez-Riverol et al., 2018) partner repository with the dataset identifier: PXD017118. These unique proteins were assigned to five categories, *viz*., energy metabolism, nucleotide processing, biotic and abiotic stress response, response to pathogen invasion, protein processing, and others (miscellaneous proteins for which one specific function could not be assessed). No statistically significant differences were found when comparing the percentage of proteins in each category present in the both plant extracts (**Supplementary Figure 2**).

### Virulence of *D. solani* Tn5 Mutants

#### Ability of *D. solani* Mutants to Macerate Potato Tuber Slices and Chicory Leaves

**Figure 4:**
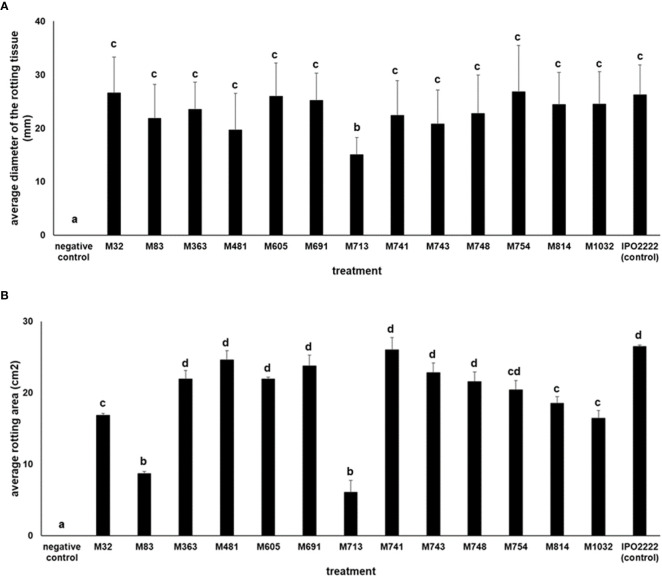
Ability of *D. solani* Tn5 mutants to cause maceration (rotting) of potato tuber slices **(A)** and chicory leaves **(B)**. Determination of the average diameter of the rotting potato tuber tissue **(A)** (in mm) was done after 72-h incubation at 28°C in a humid box. Per mutant three individual potato tuber slices, each containing three wells inoculated with bacterial suspensions in two independent experiments were used to assess the maceration ability of each individual Tn5 mutant. Per experiment, the results of nine independent measurements of rotting tissue diameters (9 biological replicates, n = 9) in two independent experiments (n = 18) were used to assess the maceration ability of each individual Tn5 mutant. The pair-wise differences were obtained using the *t*-test. *D. solani* strain IPO2222 was used as a positive control and the potato slices inoculated with sterile Ringer’s buffer served as a negative control. Determination of the average area of chicory leaf rotting **(B)** (in cm^2^) was done after 48-h incubation at 28°C in a humid box. Per mutant five individual chicory leaves (5 biological replicates, n = 5) in two independent experiments (n = 10) were used to assess the maceration ability of each individual Tn5 mutant. The pair-wise differences were obtained using the *t*-test. *D. solani* strain IPO2222 was used as a positive control and chicory leaves inoculated with sterile Ringer’s buffer served as a negative control. Values followed by identical characters are not significantly different (*p* = 0.05).

The 13 *D. solani* mutants were tested in potato tuber and in chicory leaf assays for their ability to macerate plant tissue. All mutants exhibited the ability to macerate plant tissues (either potato or chicory leaves, or both). Mutant M713 conferred significantly lower maceration of both potato tuber slices and chicory leaves (**Figure 4**) compared to the wild-type strain. Additionally, mutants M83, M713, and M1032 also caused reduced symptoms on chicory leaves compared to the wild-type strain (**Figure 4**).

#### Ability of Mutants to Cause Symptoms on *S. tuberosum* and *S. dulcamara* in Tissue-Cultured Plants

**Figure 5:**
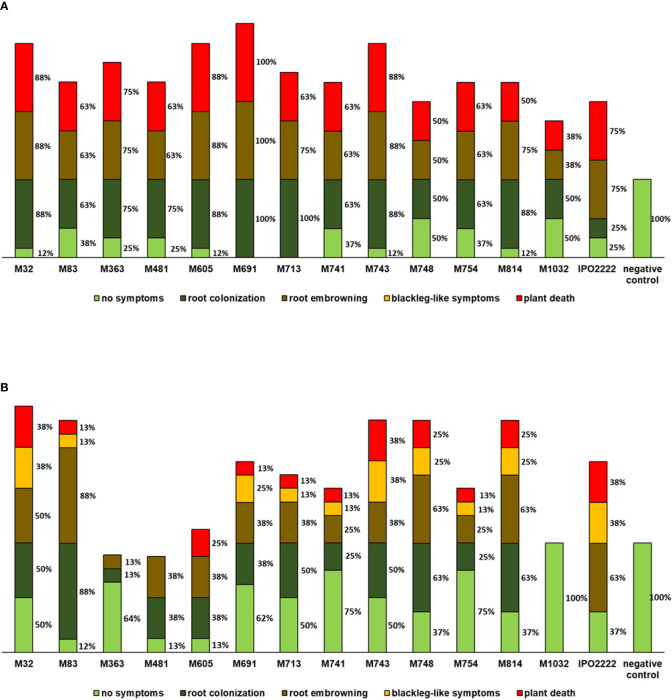
Ability of *D. solani* Tn5 mutants to cause symptoms in the culture-tube grown *S. tuberosum* **(A)** and *S. dulcamara* **(B)** plants. Disease symptoms observed after inoculation of *S. tuberosum* and *S. dulcamara* plants, shown as percentage of (affected in a respective symptom class) plants per treatment. Inoculated plants were grown *in vitro* under growth chamber conditions at 22°C and 16/8-h photoperiod. In replicated experiments 20 plants (per Tn5 mutant and plant species tested) were used and the experiment was repeated one time with the same setup (n = 40 per plant species). Plants were evaluated for symptom development at 14 days post inoculation. The sum of percentages does not give 100% as often several different classes of symptoms were observed per analyzed plant.

Selected *D. solani* Tn5 mutants were tested for their ability to cause symptoms in *S. tuberosum* and *S. dulcamara* plants grown in culture tubes. *S. tuberosum* plants inoculated with *D. solani* IPO2222 and Tn5 mutants, exhibited three distinct classes of symptoms (i) colonization of roots visualized as increase of turbidity around the roots, (ii) browning of roots obvious as a change of root color from pale white/yellowish to brown, and (iii) death of the plant with the whole plant being macerated (**Figure 5A**). Likewise, *S. dulcamara* plants displayed these classes of symptoms as well as another class: (iv) development of typical blackleg-like symptoms (blackening of the stem) in the majority of plants (**Figure 5B**). All 13 mutants caused systemic infection of *S. tuberosum* leading to severe disease symptoms including plant death in a number of plants (**Figure 5A**). Eight Tn5 mutants (*viz*., M32, M363, M481, M605, M691, M713, M743, and M814) were equally or more aggressive that the wild-type strain, whereas virulence of the five other mutants (M83, M741, M748, M754, and M1032) was reduced on *S. tuberosum*. Contrary, on *S. dulcamara* only 3 mutants (M83, M748, and M814) expressed comparable, or higher than the wild-type strain, ability to cause infection symptoms and the virulence of nine other mutants was heavily reduced (**Figure 5B**). What is more, the mutant M1032 was unable to systemically infect *S. dulcamara* plants and cause symptoms. Mutant M1032 had decreased virulence in *S. tuberosum* plants and was not virulent in *S. dulcamara* in repeated experiments. Mutants M363 and M754 exhibited a significant decrease in the ability to cause symptoms in *S. dulcamara* (**Figure 5B**).

## Discussion

Although successful colonization of plant hosts, often coupled with development of disease symptoms, is crucial for plant pathogens to complete their life cycles (Romantschuk, 1992), knowledge of the molecular mechanisms governing the colonization of plants by bacterial pathogens remains incomplete (Rovira, 1965; Sequeira, 1984). There is only limited information concerning the colonization of potato, the primary host of *D. solani* (Czajkowski et al., 2010a; Czajkowski et al., 2010b), and there is little information present in the literature of how the bacterium interacts with alternative hosts (Ma et al., 2007). This study has determined the extent to which of traits needed to colonize two host plants overlap.

In this study, we used a random Tn5-based reporter transposon mutagenesis strategy to identify those *D. solani* transcriptional units whose transcription was responsive to either a crop or weed plant host (*S. tuberosum* or *S. dulcamara¸* respectively) as well as any tissue-specific (leaf, stem, tuber, or roots) expression they might have. With this approach, we examined the hypothesis that the initial host recognition step is crucial for *D. solani* to differentiate between primary and alternative (weed, secondary) plant hosts during infection.

While the expression of a large percentage of *D. solani* transcriptional units were interrogated with our Tn5-*gusA* reporter system, we were surprised to find that only about 2.1% of the mutants exhibited strict plant tissue-dependent regulation of transcription. The low proportion of genes/operons that were specifically up-regulated in the presence of *S. tuberosum* and/or *S. dulcamara* tissues may suggest that for the successful initial contact of *D. solani* with any plant host substantial global reprograming of the *D. solani* metabolism is not required. In *D. dadantii*, a bacterium closely related to *D. solani*, the initial step of infection of *Arabidopsis thaliana* is characterized by the latent (symptomless) multiplication of the pathogen inside tissues (Lebeau et al., 2008; Chapelle et al., 2015). Similarly, in a model of the infection process based on transcriptomic data, Jiang and co-workers hypothesized that the majority of transcripts encoding virulence factors in *D. dadantii* were coordinately expressed no earlier than about 12 h after inoculation of *S. tuberosum*, while the initial symptoms appeared only after 24 to 48 h (Jiang et al., 2016). A delay in expression of virulence traits would be beneficial for *D. solani* as well as for other SRPs, since the pathogen needs to achieve a sufficiently high population size to establish a successful infection in the host (Nasser et al., 2013).

In this study, the procedure that we used to identify bacterial transcriptional units up-regulated *in planta* was rather conservative. Not only did we select mutants that did not exhibit any GUS expression *in vitro*, but this step was performed using a minimal medium that does not support the growth of auxotrophic mutants. Thus, only prototrophic *D. solani* mutants were tested in this study. This step would have eliminated mutants in genes/operons having some basal level of expression, yet which might exhibit even higher levels of expression *in planta*. By employing such a strict plant-dependent expression pattern to identify candidate transcriptional units in this study, we aimed to strongly enrich for those genes most likely to contribute to virulence to plants, which Tn5 insertions would result in decreased virulence phenotype in one or the other of both plant hosts. A recent competition approach in *D. dadantii* strain 3937 during infection of chicory revealed the importance of bacterial genes engaged in amino acid and nucleotide biosynthetic pathways, which would be expressed *in vitro*, to its fitness in this plant (Royet et al., 2019). Several genes in *P. brasiliense* involved in amino acid uptake were up-regulated during infection of potato (Bellieny-Rabelo et al., 2019). Our stringent experimental conditions may explain the relatively low number of plant tissue–specific regulated genes found in this study; however, the genes that were found were more likely to contribute to the differential fitness and virulence in the two plant hosts analyzed.

Current knowledge about the genes induced during the interaction of *Dickeya* spp. with their plant hosts have come mostly from studies of *D. dadantii* strain 3937 (Charkowski et al., 2012; Reverchon and Nasser, 2013). It is noteworthy that the low fraction of up-regulated genes of *D. solani* found in this study is about 3.5 times higher than that reported previously for *D. dadantii* strain 3937 using an IVET-leaf array which had found that only ca. 0.61% of the genes were up-regulated in the presence of spinach tissues (Yang et al., 2004). The fraction of *D. solani* genes that were up-regulated *in planta* in our study is, however, similar to the fraction (ca. 2.5%) of *D. dadantii* genes up-regulated in the initial steps of colonization of *Arabidopsis thaliana* leaves (Chapelle et al., 2015). In contrast, using genomics, metabolomics and transcriptomics, des Essarts and co-workers showed that ca. 17% of *D. dianthicola* and ca. 24% of *D. solani* genes were differently expressed in macerated potato tuber tissue in comparison to axenic cultures in rich medium (des Essarts et al., 2019). It seems clear that both the aim of the study of de Essarts and co-workers and methodological differences accounted for the variations in the observed frequencies of plant induced transcriptional units. Furthermore, although *D. dadantii* and *D. solani* share many genes, the genome of *D. solani* contains several hundred genes absent in *D. dadantii* strain 3937 (Garlant et al., 2013; Pedron et al., 2014). In addition to the distinct characteristics, these *Dickeya* spp. may possess, they may also differ in their responsiveness to plant tissue. Such a difference has been observed earlier in a comparative study of gene expression in *D. solani* and *D. dianthicola* during potato tuber infection (des Essarts et al., 2019).

Substantial quantitative differences in plant inducibility were observed in mutants of *D. solani*. While 210 mutants with some apparent induction in plant tissues were observed, the screen resulted firstly in bacterial mutants in which expression of the mutated gene was induced exclusively by *S. tuberosum* (95 genes, 45%) or *S. dulcamara* (52 genes, 25%) tissues. Secondly, from all the 210 mutants screened with plant tissues, 63 (30%) *D. solani* Tn5 mutants, however, showed up-regulation of the reporter gene expression by the presence of both plant species (**Figure 1**). Surprisingly, no obvious differences were found in the proportions of *D. solani* mutants that were up-regulated in the presence of *S. tuberosum* or *S. dulcamara* tissues. Of the 210, *D. solani* mutants that were identified as having elevated GUS reporter gene activity in the presence of either *S. tuberosum* or *S. dulcamara* in the initial screening steps, more than 50% of the mutants were up-regulated in the presence of stems derived from *S. tuberosum* (64%) and *S. dulcamara* (67%) and only a very low fraction (less than 15%) were up-regulated exclusively in the presence of either *S. tuberosum* and/or *S. dulcamara* roots (**Figure 2**). Such a result is predictable given that *D. solani* causes typical and severe blackleg symptoms in stems of field-growing potato plants (Toth et al., 2011) as well as in culture tube-grown *S. dulcamara* (Fikowicz-Krosko and Czajkowski, 2017b). In contrast, the infection of leaves and roots of growing plants with SRP often occurs without manifestation of rotting (Pérombelon, 2002; Toth et al., 2003; Czajkowski et al., 2010a). The rotting caused by SRP in field-grown potato plants tends to occur in the above-ground parts of stems even though the bacteria are often systemically distributed throughout xylem vessels of roots, leaves, and stolons (Czajkowski et al., 2010b). SRP may enter the plant though leaves and roots but stem infection appears to be the most efficient means to invade their hosts as well as to exhibit full virulence (Czajkowski et al., 2010a; Reverchon and Nasser, 2013).

The selected 13 bacterial mutants also exhibited a notable plant-dependent gene expression when analyzed with qRT-PCR. It has to be pointed out here, however, that the results obtained in the GUS assay with the Tn5 mutants and the results of the qRT-PCR analyses with the wild-type IPO2222 strain were not always convergent. The same problem was reported by others (e.g., Coutinho et al., 2015) and part of these differences in apparent inducibility could be explained by different methodological approaches themselves that were used; it is difficult to compare the induction patterns observed in the GUS assay used in screening of the mutants and the qRT-PCR performed on the genes identified in the wild-type background. Inactivation of a gene in the insertional mutants could influence its own expression as well as the expression of the operon in which the gene is located due to the polarity. For example, a mutation in a metabolic pathway resulting in a metabolic block could cause accumulation of an inducer or could limit the availability of an inducer. Moreover, the result of the GUS assay depends on the rate of protein synthesis, the stability of the GUS protein and its enzymatic activity within the 48-h assay period (initial qualitative visual screening of Tn5 mutants in presence of plant tissues). All these factors lead to the accumulation of the GUS product. In turn, the qRT-PCR method evaluates the abundance of a transcript at a given time. Thus, the observed result in the GUS quantitative assay was based on enzyme accumulation during bacterial growth, while qRT-PCR result reflected an instantaneous bacterial state at a given assay time. Likewise, it is possible that many plant-regulated genes in *D. solani* are controlled by so-called short lived transcripts (Heroven et al., 2017) and, again, these genes could be found differentially induced as measured by both methods (GUS expression and qRT-PCR).

Among the 13 *D. solani* transcriptional units whose expression was found to be up-regulated in the presence of plant tissues, the majority were induced in both *S. tuberosum* and *S. dulcamara*. Thus, these units did not exhibit host-dependent induction of transcription. However, the 13 other showed clear tissue-dependent induction in a given host as demonstrated both by the GUS assay and qRT-PCR. Of the 13 bacterial transcriptional units analyzed in this study, 5 were predicted to be transcribed as individual genes and 5 other (insertions: *d83*, *d363*, *d713*, *d748*, and *d1032*) were predicted to be situated inside operons. It cannot be excluded therefore that the observed phenotypes of the Tn5 mutants M83, M363, M713, M748, and M1032 resulted not from inactivation of a single gene but rather from inactivation of the operon inside which the gene was located. In *E. coli* and *Klebsiella pneumoniae*, the Tn5 was reported to influence the transcription of the genes localized downstream to its insertion site in the operon (Merrick et al., 1978; de Bruijn and Lupski, 1984). Although polarity effects of Tn5 transposition in *Dickeya* spp. has not been widely characterized to date (Chatterjee et al., 1983) and no knowledge exists about such transposon insertion effects in *D. solani*, it should be assumed that the introduction of the Tn5 transposon into IPO2222 chromosome will impact the transcription not only of the targeted, Tn5-disrupted gene but also may affect the transcription of the genes localized in the downstream of the insertion in the same operon. The knowledge of molecular basis of *D. solani* interactions with plant tissues is scarce. As the genes in operons are in majority functionally related to each other and regulated in the coordinate manner (Osbourn and Field, 2009) it is interesting to see that in *D. solani* IPO2222 at least several of them, as evidenced in this study, are also transcriptionally differentially induced by the presence of plant tissues derived from a primary as well as a secondary/alternative plant host.

Two main types of induction patterns were observed in our study. Firstly, the potato tuber tissue was the most effective inducer and the other plant tissues had no or little effect on expression of four transcriptional units (*ds83*, *ds481*, *ds713*, and *ds743*). Secondly, nine other units were induced by different tissues of both plant species but not by the potato tuber tissue. These data indicate a clear difference in the induction properties of the tuber tissue in comparison to stem, leaf or root tissues of a host plant. Such a difference could result either from: (i) differences in the composition of inducing plant compounds in various tissues, (ii) differences in concentration of such a compound between plant tissues, or (iii) presence of repressing conditions in one but not the other tissues. Indeed, the induction of bacterial genes in the presence of a complex material, such as plant tissues, could include both synergistic and inhibitory effects between molecules and conditions sensed by the bacterial cells (Hugouvieux-Cotte-Pattat et al., 1996). The observed induction patterns suggest that *D. solani* is able to distinguish the host tissues at the early stage of contact and, accordingly, adjusts its gene expression.

The phenotypic analysis of the 13 selected mutants indicates that one of the most frequently observed phenotypes (present in six mutants: M363, M481, M691, M743, M748, and M754) was a lack of chemotactic response to extracts of *S. tuberosum* or *S. dulcamara*. Notably, two mutants (M481 and M754) were unable to sense compounds present in both plant extracts. These complex plant extracts share common compounds as evidenced by mass spectrometry analysis but also contain unique compounds present in only one of the screened plant hosts. With the use of mass spectrometry, we indeed identified 200 unique proteins present in *S. dulcamara*. These unique proteins may contribute to the apparent resistance of the plant against *D. solani* infections. No major difference was, however, found when comparing the percentage of proteins in each category present in the both plant extracts. This suggest that the induction of IPO2222 genes/operons by the presence of plant tissues may be triggered by the occurrence of an individual plant species-specific protein (and not a specific class of proteins) present in one but absent in the other plant species extracts. As the extracts did not contain any plant proteins already reported to cause the induction of transcription in *Dickeya* spp. (and *D. solani*), the composition of the *S. tuberosum* and *S. dulcamara* extracts should be further in detail evaluated.

The severity of symptoms development in culture-tube grown plants as well as the frequency of symptoms in a given class differed largely between *S. tuberosum* and *S. dulcamara*. The 8 from the 13 Tn5 mutants (61.5%), interrogated for the virulence on *S. tuberosum* expressed disease symptoms as vigorously as the wild-type strain or were even more virulent causing symptoms in the high number of plants, whereas on *S. dulcamara* only 3 mutants (23%) from the pool of 13 mutants screened shown aggressiveness higher or similar to the aggressiveness of the wild-type strain (**Figures 5A, B**). Likewise, the mutants M481 and M754 exhibited decreased virulence in *S. dulcamara*, but virulence comparable to the virulence of *D. solani* IPO2222 in *S. tuberosum* and the mutant M691 exhibited decreased virulence in *S. dulcamara* but was more aggressive than the IPO2222 wild-type strain in *S. tuberosum*. Such differences suggest that *D. solani* is able to more easily attack *S. tuberosum* than *S. dulcamara* and that possibly some bacterial transcriptional units are needed exclusively to infect other hosts than potato (e.g., *S. dulcamara*). This is in line with our earlier studies in which *S. dulcamara* plants harboring relatively high pathogen levels remained symptomless (Fikowicz-Krosko and Czajkowski, 2017b), whereas the same amount of *D. solani* inoculum caused severe rotting of potato plants. Moreover, the gene disrupted in mutant M754, which is induced by all plant tissues except tubers, encodes a potential chemotaxis receptor. Chemotaxis is recognized as a factor dictating virulence of many plant pathogenic bacteria (Vande Broek and Vanderleyden, 1995; Brencic and Winans, 2005) including *D. dadantii* (Antúnez-Lamas et al., 2009) and *D. solani* (Golanowska et al., 2018). Notably, several genes encoding chemotaxis receptors were differentially expressed during potato infection by *D. solani* in a recent report (des Essarts et al., 2019). However, a direct linkage between chemotaxis and virulence is difficult to establish because plant extracts may contain several chemoattractants. Furthermore, the genomes of plant pathogenic bacteria encode many chemotaxis receptors having functional redundancy (Matilla and Krell, 2017). For example, 43 predicted chemotaxis receptors are present in *D. solani* strain IPO2222 (Khayi et al., 2016).

The plant-induced genes found here include several involved in the assimilation of carbon sources as well as encoding porins present in the outer membrane that facilitate uptake of two types of oligosaccharides released during pectin degradation, *viz*., oligogalacturonides (gene *kdgN*, in mutant M814) and oligogalactans (gene *ganL*, in mutant M691) (Condemine and Ghazi, 2007; Delangle et al., 2007). Genes encoding the enzyme involved in galactarate assimilation (*garD*, in mutant M32), and an element of an ABC transporter involved in phosphate entry (*pstB*, in mutant M748) are also induced by plant tissues. In our earlier studies (Czajkowski et al., 2017; Lisicka et al., 2018), we identified some temperature and hypoxia-regulated genes in *D. solani* that influenced the virulence of this strain and found many to be involved in a primary bacterial metabolism. This supports earlier speculations that SRP virulence results from a complex network of interactions involving fundamental bacterial metabolism as well as production and secretion of factors that contribute strictly in virulence (Salmond, 1994; Charkowski, 2007; Hugouvieux-Cotte-Pattat, 2016). The fact that several integral membrane proteins as well as proteins affecting the envelope structure were encoded by the plant-induced genes (mutants M83, M691, M713, M754, and M814) confirms the major role of the bacterial envelope in the exchanges between bacteria and their environment and, in particular, during the interaction of *D. solani* with plant tissues.

Genes defined in two insertions (*ds83* and *ds713*) that are induced exclusively in the presence of potato tuber tissues are in close proximity to each other in the genome of *D. solani* strain IPO2222. They encode GDP-l-fucose synthase and GDP-mannose 4,6-dehydratase, which are involved in the biosynthesis of exopolysaccharide (EPS) in *Pectobacterium carotovorum*. A *P. carotovorum* GDP-l-fucose synthase knockout mutant had reduced virulence in both carrot and celery (Islam et al., 2019). Likewise, EPS was considered necessary for attachment of *D. dadantii* cells to plant surfaces during infection (Condemine et al., 1999; Reverchon and Nasser, 2013). In our study, *D. solani* IPO2222 mutants M83 and M713 also had a reduced ability to macerate both potato tuber slices and chicory leaves. Such results highlight the importance of EPS production during SRP interaction with plant hosts (Leigh and Coplin, 1992).

*D. solani* is an emerging pathogen with the ability to constrain agricultural production worldwide. The identification of candidate *D. solani* genes important in the early colonization of primary and alternative host plant can help in understanding how necrotrophic bacterial pathogens recognize plants and alter their metabolism and express virulence traits to be successful pathogens. This, in turn, may result in development of novel means of control and prevention in the future. Screening of the Tn5 mutant library for mutants affected in the colonization process is likely to reveal additional virulence factors.

## Data Availability Statement

All datasets presented in this study are included in the article/supplementary material.

## Author Contributions

RC: conceptualization of the study, formal analysis, funding acquisition, resources, supervision, visualization, writing, reviewing, and editing of the manuscript, analyses of the qRT-PCR results, assessment of colony morphology of Tn5 mutants, determination of the Tn5 mutants average generation time in rich and poor media, assessment of the growth of Tn5 mutants in the presence of plant extracts, assessment of chemotaxis of Tn5 mutants, phenotypic characterization of Tn5 mutants with BIOLOG microarrays, phenotypic characterization of Tn5 mutants with biochemical and plate assays, assessment of biofilm formation, assessment of growth of Tn5 mutants under anaerobic conditions, assessment of AHLs production, and statistical analyses. JF-K: transposon mutagenesis of *D. solani* IPO2222, verification of Tn5 mutants with PCR and CVP plating, selection of *S. tuberosum* and *S. dulcamara* induced Tn5 mutants, propagation of the *in vitro* plants, qualitative and quantitative GUS expression analyses, assessment of colony morphology of Tn5 mutants, determination of the Tn5 mutants average generation time in rich and poor media, phenotypic characterization of Tn5 mutants with BIOLOG microarrays, phenotypic characterization of Tn5 mutants with biochemical and plate assays, assessment of host colonization and virulence of Tn5 mutants on *in vitro* plants, assessment of ability of Tn5 mutants to macerate potato and chicory tissues, identification and analysis of the Tn5 insertion sites in bacterial genomes, identification of the functions of Tn5-distrupted genes. TM: analyses of the biochemical pathways and cellular networks affected by Tn5 insertions in bacterial mutants, resources, and software. LR: bioinformatic analyses of the Tn5 mutant genomes, identification and analysis of the Tn5 insertion sites, identification of the functions of Tn5-distrupted genes, resources, and software. PC: mass spectrometry analyses of the *S. tuberosum* and *S. dulcamara* extracts, resources, and software. SJ: Southern blotting analyses of the Tn5 insertions in the genomes of bacterial mutants, resource, and editing of the manuscript. MK-M: phenotypic characterization of the *D. solani* Tn5 mutants, data analyses, visualization, and resources. MR: assessment of cell morphology of Tn5 mutants with transmission electron microscopy, resources, software. NH-C-P: conceptualization of the study, writing, reviewing, and editing of the manuscript.

## Funding

The work was financially supported by the National Science Centre, Poland (Narodowe Centrum Nauki, Polska), *via* a research grant SONATA 8 (2014/15/D/NZ9/00605) to RC, by Polish Ministry of Science and Higher Education (Ministerstwo Nauki i Szkolnictwa Wyższego, Polska) funds DS 531-M034-D786-19 to SJ and by Polish Ministry of Higher Education (Ministers two Nauki i Szkolnictwa Wyższego, Polska) funds DS 531-N104-D800-20 to RC.

## Conflict of Interest

The authors declare that the research was conducted in the absence of any commercial or financial relationships that could be construed as a potential conflict of interest.
