# Supplementary material for: Genome-Wide Identification of Dickeya solani Transcriptional Units Up-Regulated in Response to Plant Tissues From a Crop-Host Solanum tuberosum and a Weed-Host Solanum dulcamara
Source: Front Plant Sci. 2020 Sep 2;11:580330. doi: 10.3389/fpls.2020.580330 (PMC7492773; doi:10.3389/fpls.2020.580330)

Supplementary Material for:

Genome-wide identification of *Dickeya solani* transcriptional units upregulated in response to plant tissues from a crop-host *Solanum tuberosum* and a weed-host *Solanum dulcamara*

Robert Czajkowski [1] *, Jakub Fikowicz-Krosko [1], Tomasz Maciag [2], Lukasz Rabalski [3], Paulina Czaplewska [4], Sylwia Jafra [2], Malwina Richert [5], Marta Krychowiak-Maśnicka [1] and Nicole Hugouvieux-Cotte-Pattat [6]

[1] Division of Biologically Active Compounds, [2] Division of Biological Plant Protection, [3] Division of Recombinant Vaccines, [4] Laboratory of Mass Spectrometry - Core Facility Laboratories, Intercollegiate Faculty of Biotechnology UG and MUG, University of Gdansk, Antoniego Abrahama 58, 80-307 Gdansk, Poland

[5] Laboratory of Electron Microscopy, Faculty of Biology, University of Gdansk, Wita Stwosza 59, 80-308, Gdansk, Poland

[6] Microbiology Adaptation and Pathogenesis, CNRS UMR5240, University of Lyon, University Claude Bernard Lyon 1, INSA Lyon, Villeurbanne, France

***Correspondence:**
Robert Czajkowski
robert.czajkowski@biotech.ug.edu.pl

Keywords: potato, bittersweet nightshade, Tn5, mutagenesis, alternative plant host, *Erwinia chrysanthemi*

SUPPLEMENTARY MATERIALS AND METHODS

Assessment of colony morphology of *D. solani* Tn5 transposon mutants

Selected *D. solani* Tn5 disruption mutants were analyzed for changes in colony morphology as previously described (Lisicka et al., 2018). The morphology of bacterial colonies was analyzed using a Leica MZ10F stereomicroscope at 10 x and 40 x magnification coupled with a Leica DFC450C camera system (Leica). At least ten images were taken of each mutant and wild type strain IPO2222, used as a control.

Assessment of cell morphology of *D. solani* Tn5 transposon mutants

The morphology of bacterial cells was assessed using transmission electron microscopy (TEM). TEM analyses were performed by the Laboratory of Electron Microscopy, Faculty of Biology, University of Gdansk, Poland. Bacteria were adsorbed onto carbon-coated grids (Sigma-Aldrich) stained with 1.5% uranyl acetate and directly examined with an electron microscope (Tecnai Spirit BioTWIN, FEI) as described previously (Czajkowski et al., 2017). At least ten images were taken of each mutant and the wild type strain to estimate cell diameter.

Determination of the average generation time of *D. solani* Tn5 mutants in rich and minimal media

To determine whether the Tn5 insertions affected the generation time of the mutants, the growth of the selected *D. solani* Tn5 mutants was assessed in TSB (rich medium) and M9 supplemented with 0.4% glucose (minimal medium) at 28 °C for 16 h as previously described (Czajkowski et al., 2017). Briefly, overnight bacterial cultures with density of ca. 10^9 cfu ml^{-1} in TSB or M9 + 0.4% glucose were diluted 50-fold in the same fresh medium. One hundred microliters of diluted bacterial culture was aseptically transferred to the wells of 96-well microtiter plates (NEST) and sealed with optically clear sealing tape (Sarstedt) to prevent desiccation of bacterial culture. Growth rate was determined by measuring the optical density (OD) (λ=600 nm) every 30 min in an Epoch2 Microplate Spectrophotometer (BioTek) for 16 h as previously described (Lisicka et al., 2018). The growth of each mutant was analyzed in duplicate and the results were averaged. Each 96-well plate contained two negative (sterile growth medium) and two positive (wild type *D. solani* IPO2222 culture) wells as controls. The experiment was independently repeated once and the results were averaged. The average generation time was calculated using Doubling Time calculator (parameters: C0= 3 h, Ct=7 h, t=4 h) (http://www.doubling-time.com/compute.php) (Roth, 2006).

Growth of *D. solani* Tn5 mutants *in vitro* in the presence of *S. tuberosum* and *S. dulcamara* extracts

The growth of selected Tn5 mutants was assessed in M9 medium supplemented with 0.4% glucose and 5% plant (*S. tuberosum* or *S. dulcamara*) extracts at 28 °C for 16 h. The culture-tube grown *S. tuberosum* or *S. dulcamara* plants (including roots), propagated *in vitro* as described above, were collected from the agar medium, briefly washed in sterile demineralized water to remove agar and weighted. The plants were crushed for 1-2 min in BIOREBA extraction bags (BIOREBA) after adding twice the weight of 1/4 Ringer's buffer (Merck) containing 0.02% diethyldithiocarbamic acid (DIECA; Sigma-Aldrich) as an oxidant (Perombelon and van Der Wolf, 2002; Czajkowski et al., 2010b). The plant extracts were sterilized using 0.22 µm syringe filters (VWR). The plant extracts were used immediately or stored at -20 °C for further use. Bacterial growth was analyzed by measuring the optical density at 600 nm as described above. The experiment was repeated once and the results were averaged.

Chemotaxis towards *S. tuberosum* and *S. dulcamara* plant extracts

Selected Tn5 mutants were tested for their chemotactic response towards *S. tuberosum* and *S. dulcamara* extracts using a modified disk diffusion assay (de Weert et al., 2002). Briefly, bacteria were grown for 16 h in TSB at 28°C with vigorous shaking (150 rpm) to reach densities of ca. 10^9 cfu ml^{-1}. The cells were then collected by centrifugation (6000 x g), washed two times with Ringer's buffer and resuspended in 10 ml of 0.05% sterile agarose (Sigma-Aldrich) in demineralized water to ca. 10^{10} cfu ml^{-1}. The resulting suspensions were individually transferred to sterile Petri dishes (10 cm diameter, Sarstedt), where the suspensions formed an approx. 3-4 mm-thick layer. Ten microliter drops of sterile *S. tuberosum* or *S. dulcamara* extracts were placed on sterile 0.5 cm-diameter paper disks (filter grade 3, Whatman) and dried. The disks were then placed at the center of the Petri dish containing bacterial suspensions in 0.05% agarose. After 2 h incubation at room temperature, the plates were inspected for the appearance of a clear zone surrounding the paper disks, indicating an attraction of bacterial cells by the plant extract on the disks (chemoattraction – positive reaction). The diameter of the zone was measured per each tested Tn5 mutant. *D. solani* strain IPO2222 was used as a positive control and Whatman paper disk without plant extract was used as a negative control. The experiment was repeated once with the same setup and the results were averaged.

Phenotypic characterization of *D. solani* Tn5 mutants with biochemical and plate assays

Selected *D. solani* Tn5 mutants were evaluated for their ability to grow with mannose, maltose or galactaric acid as a sole carbon source. The growth of Tn5 mutants was assessed at 28°C for 16 h in M9 medium supplemented with 0.4% of a given individual carbohydrate (mannose, maltose or galactaric acid). The doubling time was assessed as above.

Biofilm formation assay

Selected *D. solani* Tn5 mutants were evaluated for their ability to form biofilms as previously described (Nykyri et al., 2013). Briefly, 10 µl of an overnight culture in TSB at 28°C with shaking (150 rpm) was inoculated into 400 µl of M9 minimal medium supplemented with 0.4% glycerol in polypropylene 1.5 ml Eppendorf tubes. Tubes were incubated without agitation at 22 °C for 72 h and 28°C for 24 h. Cells adhering to the inner tube surfaces were stained with 600 µl of 1% (w/v) crystal violet solution for 20 min at room temperature. Tubes were then washed three times with distilled water, and air-dried. Six hundred µl of 96% ethanol (Sigma) was added to each tube to extract crystal violet absorbed by the biofilm. The concentration of crystal violet was quantified in 100 µl aliquots in a 96-well plate at 565 nm. Assay on each mutant was repeated once.

Growth under anaerobic conditions

Growth under anaerobic conditions was assessed as previously described (Czajkowski et al., 2012) with minor modifications. Bacteria grown in TSB (WT *D. solani* IPO2222) or in TSB supplemented with 50 µg ml^{-1} neomycin (Tn5 mutants) at 28°C for ca. 16 h were diluted 1:50 in the same medium but not supplemented with the antibiotic. Two ml of liquid paraffin (Sigma) was added to the top of the cultures that were then kept at 28°C without agitation for 12-48 h. Tubes were inspected daily for turbidity by eye, and after 48 h the turbidity of the Tn5 mutant and *D. solani* wild type cultures was measured spectrophotometrically (λ= 600 nm). The experiment was repeated once and the results were averaged.

Production of AHLs by *D. solani* Tn5 mutants

Selected *D. solani* Tn5 mutants and the IPO2222 wild type strain were tested for their ability to produce quorum sensing signal molecules: acyl-homoserine lactones (AHLs). Production was assessed with the AHL reporter strain *Chromobacterium violaceum* CVO26 (Latifi et al., 1995) using a previously described protocol (Czajkowski et al., 2012). The experiment was repeated once.

Ability of *D. solani* Tn5 mutants to macerate potato tuber slices and chicory leaves

Potato tubers of cv. Bryza and witloof chicory (*Cichorium intybus* L.) heads were obtained locally in Gdansk, Poland. For each mutant, an individual potato slice from three different potato tubers and five individual chicory leaves from five different plants were inoculated and disease assessed was as described before (Czajkowski et al., 2012; Krzyzanowska et al., 2012). Wild type *D. solani* IPO2222 was used as a positive control and the negative control was sterile demineralized water. Two replicates were assessed for each mutant and the results averaged.

Analyses of the *S. tuberosum* and *S. dulcamara* extracts by mass spectrometry (MS)

MS analyses of *S. tuberosum* and *S. dulcamara* extracts were done to assess the presence and concentration of known antibacterial peptides (Parachin and Franco, 2014; Bártová et al., 2019) and other plant products (carbon and nitrogen sources) that could influence the interaction of *D. solani* wild type and Tn5 mutants with host plants.

Preparation of plant extracts

S. tuberosum or *S. dulcamara* extracts were prepared by crushing plant tissues in the presence of 1/4 Ringer's buffer. The extracts were either used immediately for mass spectrometry analyses or stored at -20 °C until needed. For the MS analyses, 1 ml of *S. tuberosum* and *S. dulcamara* plant extracts were lyophilized overnight and the resulting lyophilizates were dissolved in 400 µl of UA buffer (8 M urea in 0.1 M Tris/HCl), vortexed and sonicated for 5 minutes. Afterwards, samples were filtrated with the use a 0.22 µm syringe filter (PTFE membrane, Whatman). The concentration of proteins in the samples was measured with the use of NanoDrop and the concentrations were processed with a FASP standard protocol (Wisniewski et al., 2009) using a 10 kDa Microcon filtration device (Millipore) after addition of 2 ml of trypsin (Trypsin Gold, Promega) to each sample. The concentration of tryptic peptides was measured using a UV-spectrometer at 280 nm. Per sample a total of 30 mg of tryptic peptides was desalted on StageTips (3 layers of 3M Empore C18 exchange discs) as described earlier (Rappsilber et al., 2007) and used for LC-MS/MS analysis.

LC-MS/MS analysis of peptides and identification of proteins in the plant extracts

LC-MS/MS analyses were carried out on Triple Tof 5600+ mass spectrometer with DuoSpray Ion Source (Sciex) connected to Ekspert MicroLC200 Plus System (Eksigent, Redwood City, CA). The microLC-MS/MS system was controlled by the SCIEX Analyst TF 1.7.1 software**.** Samples were loaded onto the column using the CTC Pal Autosampler (CTC Analytics AG, Zwinger, Switzerland) with each injection of 5 µl of sample, and separated on 3C18-CL-120, 3 µm,120 Å, size 0.3 x 150 mm column (Exigent) using 60 min gradient (5-20 % B, Solvent A 0% aqueous solution | 0.1% formic acid, Solvent B 100% acetonitrile | 0.1% formic acid). Source temperature (at setpoint) was 300 °C. (Information Dependent Acquisition (IDA) Switch Criteria**:** with charge state: 2 to 5**,** which exceeds: 100 cps**,** exclude former target ions: for 5 seconds**,** Exclude former ions after 2 Repeats, Maximum number of candidate ions to monitor per cycle: 20 spectra**,** Mass Defect Filter: No, Ions Tolerance: 50.000 mDa. Include / Exclude: Dynamic Background Subtract: No, Rolling Collision Energy: Yes). Up to the 20 most intense ions in each full MS scan were fragmented and analyzed. RAW spectra files were processed with Protein Pilot (v. 4.5) with Paragon TM algorithm against *Solanum* database (Uniprot) with an automated false discovery rate. The search parameters for samples derived from *S. tuberosum* and *S. dulcamara* extracts included: instrument TripleTOF 5600, alkylation of cysteines by iodoacetamide, urea modification, trypsin enzyme digestion, ID focus on biological modifications, search effort "thorough ID", and detected protein threshold [Conf] > 10%. The mass spectrometry proteomics data were deposited to the Proteome Xchange Consortium via the PRIDE (Perez-Riverol et al., 2018) partner repository with the dataset identifier: PXD017118.

SUPPLEMENTARY TABLES

Supplementary Table 1. Predicted molecular functions of the *D. solani* IPO2222 proteins differentially expressed in contact with *S. tuberosum* and *S. dulcamara* plant tissues

No	Insertion name, Tn5 locus	Localization in the *D. solani* IPO2222 genome<br><br>(Genbank accession: NZ_CP015137.1)	Homologs in *Dickeya* spp.[A]	Putative protein interaction partners according to STRING (https://string-db.org/) [B]
1	***ds32***, *garD*	1 086 892 –1 088 481 (positive strand)	*D. chrysanthemi,*<br>*D. dadantii,*<br>*D. dianthicola,*<br>*D. fangzhongdai,*<br>*D. lacustris,*<br>*D. undicola,*<br>*D. zeae*	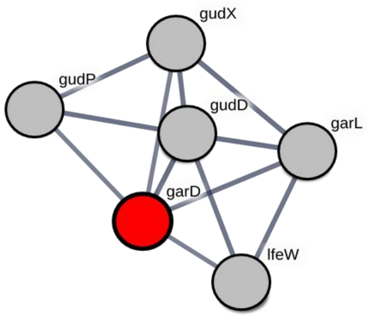<br>***gudD*** - D-glucarate dehydratase<br>***garL***- 5-keto-4-deoxy-D-glucarate aldolase<br>***gudX***- D-glucarate dehydratase-related protein<br>***gudP***- predicted D-glucarate transporter;<br>***lfeW***- mandelate racemase/muconate lactonizing enzyme family protein

**2**	***ds83***, *fcl*	1 447 537 – 1 448 475<br><br>(positive strand)	*D. dadantii,*<br><br>*D. dianthicola,*<br><br>*D. fangzhongdai,*<br>*D. undicola,*<br>*D. zeae*	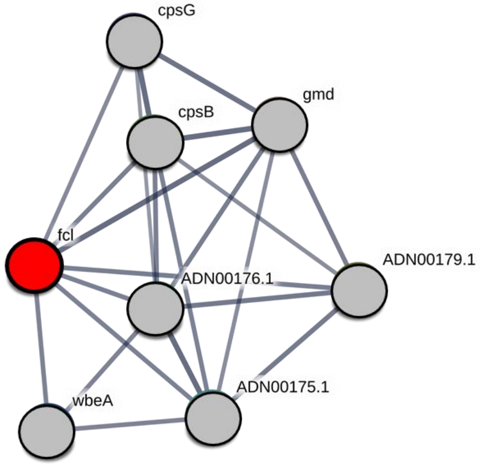<br><br>***gmd*** – GDP-mannose 4,6-dehydratase<br>***ADN00179.1***- glycosyltransferase WbpZ<br>***cpsB*** – mannose-1-phosphate guanylyltransferase<br>***ADN00176.1*** - O-antigen export system, ATP-binding protein<br>***ADN00175.1***- O-antigen export system permease protein RfbD<br>***wbeA*** - annotation not available<br>***cpsG*** - phosphomannomutase

**3**	***ds363***, *nrdD*	2 573 836 –2 575 974 (negative strand)	*D. chrysanthemi,*<br>*D. dadantii,*<br>*D. dianthicola,*<br>*D. fangzhongdai,*<br>*D. lacustris,*<br>*D. paradisiaca,*<br>*D. undicola,*<br>*D. zeae*	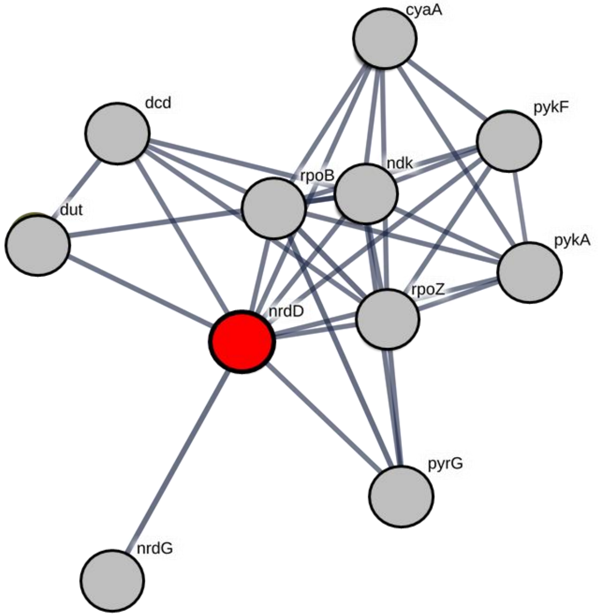<br>***nrdG*** - anaerobic ribonucleoside-triphosphate reductase-activating protein<br>***dut*** - deoxyuridine 5’-triphosphate nucleotidohydrolase<br>***pyrG*** - CTP synthase<br>***cyaA*** - protein involved in adenylate cyclase activity<br>***pykF*** - pyruvate kinase<br>***pykA*** - pyruvate kinase<br>***rpoZ*** - DNA-directed RNA polymerase subunit omega<br>***rpoB*** - DNA-directed RNA polymerase subunit beta<br>***ndk*** - nucleoside diphosphate kinase<br>***dcd*** - dCTP deaminase

**4**	***ds481***,<br>A4U42_RS1730<br>0	4 035 163 – 4 038 162<br>(positive strand)	no homologs in<br>*Dickeya* spp.	no interaction partners found
**5**	***ds605***,<br>A4U42_RS1588<br>0	3 668 240 – 3 669 208<br><br>(positive strand)	*D. chrysanthemi,*<br>*D. dadantii,*<br>*D. fangzhongdai,*<br>*D. undicola,*<br>*D. zeae*	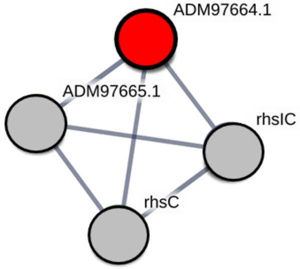<br>***ADM97665.1*** – protein with unknown molecular function<br>***rhsC*** - putative deoxyribonuclease RhsC<br>***rhsIC*** - immunity protein RhsIC
**6**	***ds691***, *ganL*	622 194 – 623 465<br>(positive strand)	*D. dadantii,*<br><br>*D. fangzhongdai,*<br><br>*D. lacustris,*<br>*D. undicola,*<br><br>*D. zeae*	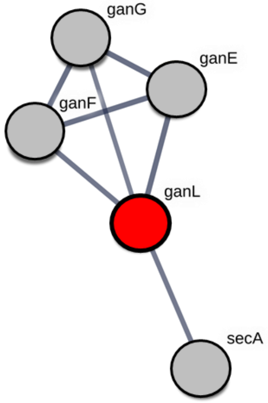<br>***ganE*** – periplasmic binding protein of galactan transport system<br>***ganF*** – galactan ABC transport system, permease component<br>***secA*** – protein translocase subunit SecA<br>***ganG*** – galactan ABC transport system, permease component

**7**	***ds713***, *gmd*	1 444 835 –1 445 953 (positive strand)	*D. chrysanthemi*,<br><br>*D. dadantii*,<br><br>*D. dianthicola*,<br><br>*D. undicola*,<br>*D. zeae*	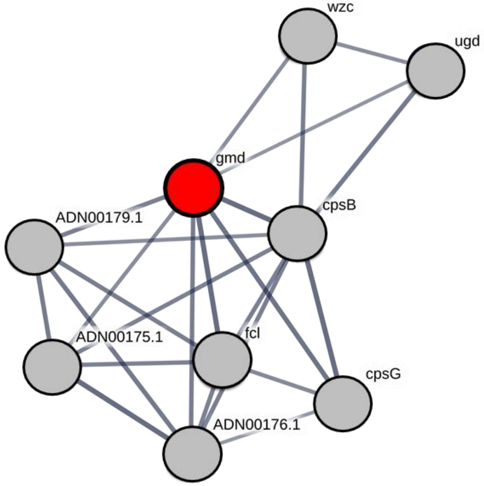<br>***fcl*** – GDP-L-fucose synthetase colanic acid biosynthesis protein WcaG<br>***cpsB*** – mannose-1-phosphate guanylyltransferase<br>***cpsG*** – phosphomannomutase<br>***ADN00176.1*** – O-antigen export system, ATP-binding protein<br>***ADN00179.1*** – glycosyltransferase WbpZ<br>***ADN00175.1*** – O-antigen export system permease protein RfbD<br>***wzc***- Tyrosine-protein kinase<br>***ugd*** - UDP-glucose dehydrogenase

**8**	***ds741***, *thiJ*	4 634 122 –4 634 907<br>(positive strand)	*D. chrysanthemi,*<br>*D. dadantii,*<br>*D. fangzhongdai,*<br>*D. lacustris,*<br>*D. zeae*	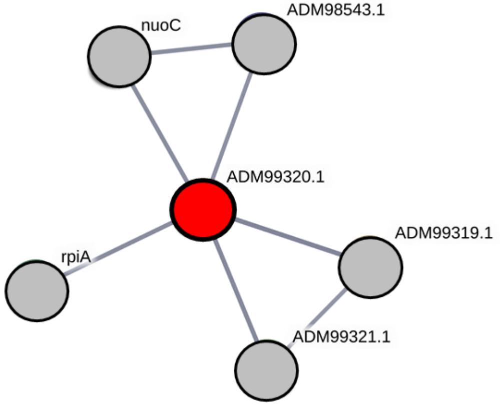<br>***ADM99319.1*** - glutathione S-transferase<br>***ADM99321.1*** – aldehyde dehydrogenase<br>***rpiA*** - ribose-5-phosphate isomerase A<br>***nuoC*** – NADH-quinone oxidoreductase subunit C/D<br>***ADM98543.1*** - PfpI family protein
**9**	***ds743***,<br>A4U42_RS1915<br>0	4 454 620 – 4 455 063<br>(positive strand)	*D. dadantii,*<br>*D. dianthicola,*<br>*D. fangzhongdai,*<br>*D. undicola*	no interaction partners found
**10**	***ds748***, *pstB*	2 129 605 –2 130 381<br>(positive strand)	*D. dadantii,*<br>*D. lacustris*	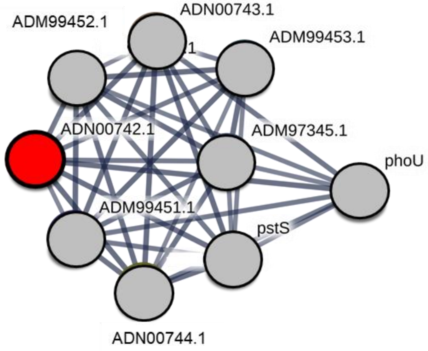<br>***ADN00743.1*** - phosphate transport system permease protein PstA

				ADN00744.1 - phosphate transport system permease protein<br>***phoU*** - phosphate-specific transport system accessory protein PhoU<br>***pstS*** - phosphate-binding protein PstS; part of the ABC transporter complex PstSACB involved in phosphate import<br>***ADM99452.1*** - protein involved in transporter activity<br>***ADM99453.1*** - phosphate ABC transporter, permease component<br>***ADM97345.1*** - phosphate-binding protein of an ABC transporter<br>***ADM99451.1*** - phosphate import ATP-binding protein PstB
**11**	***ds754***, A4U42_RS0476 5	1 126 077 –1 127 948 (negative strand)	*D. zeae*	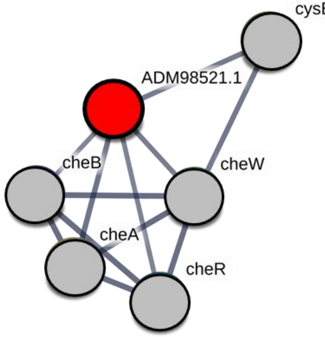<br>***cheA*** - chemotaxis protein CheA<br>***cysE*** - serine acetyltransferase<br>***cheW*** - positive regulator of CheA protein activity<br>***cheR*** - chemotaxis protein methyltransferase<br>***cheB*** - chemotaxis response regulator protein-glutamate methylesterase
**12**	***ds814***, *kdgN*	4 505 018 –4 505 743 (negative strand)	*D. chrysanthemi,*<br><br>*D. dadantii,*<br><br>*D. dianthicola,*<br><br>*D. fangzhongdai,*	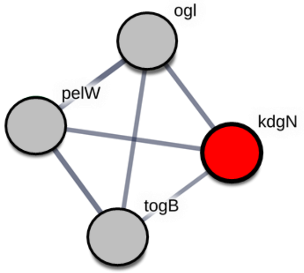

			D. lacustris,<br>*D. undicola,*<br>*D. zeae*	ogl – oligogalacturonate lyase<br>pelW – pectate disaccharide-lyase<br>togB – binding protein of the oligogalacturonide ABC transport system
**13**	***ds1032***, A4U42_RS0524 0	1 237 619 –1 238 167 (positive strand)	*D. aquatica,*<br>*D. chrysanthemi,*<br>*D. dadantii,*<br>*D. fangzhongdai,*<br>*D. paradisiaca,*<br>*D. undicola,*<br>*D. zeae*	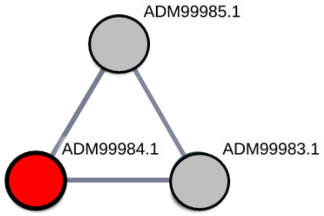<br>ADM99985.1 - glucosamine kinase GpsK<br>ADM99983.1 – protein with ATP-binding cassette

[A] – Search done with NCBI BlastP using amino acid sequence of the protein from *D. solani* strain IPO2222, cutoff was set to 70% protein identity.

[B] - Search Tool for Retrieval of Interacting Genes/Proteins v11 accessed *via* https://string-db.org/, *D. dadantii* strain 3937, closely related to *D. solani* strain IPO2222 was used as a model to assess the putative interaction partners of *D. solani* IPO2222 proteins found in this study. Only high confidence (0.700 and above) scores are shown (Szklarczyk et al., 2019), line density between the nodes indicate the strength of data support, the protein of interest is marked with a red circle and its interaction partners are marked with grey circles.

Supplementary Table 2. Phenotypes of the 13 *D. solani* Tn5 mutants found to be upregulated in the presence of *S. tuberosum* and/or *S. dulcamara* plant tissues

									**Chemotaxis to extract from:**	
**No**	**Mutant**	**Colony morphology**	**Cell morphology (TEM)**	**Generation time**	**Growth under anaerobic conditions**	**Biofilm formation**	**AHL production**	**Growth in the presence of *S. tuberosum* or *S. dulcamara* extracts**	***S. tuberosum***	***S. dulcamara***
**1**	M32	wt [A]	wt	wt	wt	wt	wt	wt	wt	wt
**2**	M83	wt	wt	wt	wt	wt	wt	wt	wt	wt
**3**	M363	wt	wt	wt	wt	wt	wt	wt	wt	–
**4**	M481	wt	wt	wt	wt	wt	wt	wt	– [B]	–
**5**	M605	wt	wt	wt	wt	wt	wt	wt	wt	wt
**6**	M691	wt	elongated cells	wt	wt	wt	wt	wt	wt	–
**7**	M713	wt	wt	wt	wt	wt	wt	wt	wt	wt

**8**	M741	wt	wt	wt	wt	wt	wt	wt	wt	wt
**9**	M743	wt	wt	wt	wt	wt	wt	wt	wt	_
**10**	M748	wt	wt	wt	wt	wt	wt	wt	wt	_
**11**	M754	wt	wt	wt	wt	wt	wt	wt	_	_
**12**	M814	wt	wt	wt	wt	wt	wt	wt	wt	wt
**13**	M1032	wt	wt	wt	wt	wt	wt	wt	wt	wt

[A] – The phenotype not significantly different from that of the *D. solani* wild type strain IPO2222

[B] – Chemotaxis not observed

Supplementary Table 3. Sequences of primers used in qRT-PCR analyses.
The qRT-PCR analyses (including primer design) were obtained commercially from Amplicon, Poland (https://en.amplicon.pl/)

No.	Insertion name	Primer *L* (left) (5'-3')	Primer *R* (right) (5'-3')	Annealing temp. [°C]	Concentration [μM]	Product length (bp.)
**1**	***ds32***	TCCGAAGTCACCGAAGTT	TGTCCAGATAATCGTCATACC A	58	0.5	115
**2**	***ds83***	ACCGTCGCCTCTTATCCTT	AGCCATACTCGCCTCCAT	58	0.5	91
**3**	***ds363***	TGCTGAACAAGGCGTTGAT	CTGAAGGCGGACGACAAT	58	1	103
**4**	***ds481***	CAGAGATGATGGCGTAGGTAT GTC	GGGAAGAGATGCGTTTGTATG TTG	58	0.5	100
**5**	***ds605***	GGCTGGCGAATGAGTTGT	GCAAGCGGATAGCGGATG	58	0.5	121
**6**	***ds691***	CAAAGCGGCGAGAAATAC	CGTTCAGATAAGAGGTGTAGA	58	0.5	97
**7**	***ds713***	TGCGGATGTTGATGCTAT	CACCAGACCATACAGTTCA	58	0.5	115
**8**	***ds741***	TGACCACCGACGAGCAGAAT	GACCAACCCTCAACACCGAAA C	58	1.0	96

**9**	***ds743***	AGTGACGGTTCTACATTTATTCT	GTCGCCTTCCATCAGTTC	58	1.0	96
**10**	***ds748***	TGGTGATCGTGACGCATAA	GGTATCGGTATCGCTGAACT	58	0.5	101
**11**	***ds754***	TTGCTGCGGATGACGACTC	GTAGTGCGGTGACGGAGATG	58	0.5	75
**12**	***ds814***	TGTTGTATTCCAGTTCGTATTCA	CTCTGCGTTACCGTCCTT	58	1.0	150
**13**	***ds1032***	CATGACGCCACTCACACC	ACCAGTCCACCACCTGTT	58	0.5	77
**14**	***lpxC***	ATGAACAGGTCGCCAATCG	AGATGATTACCGTGTGCTGAAC	58	1.0	93
**15**	***yhB***	CAGAATAAGCCAGCACAT	CGATAGACAGCCAGACAT	58	0.5	150

SUPPLEMENTARY FIGURES

Supplementary Figure 1. Genetic context of the Tn5 insertion in *D. solani* selected Tn5 mutants. The *D. solani* ORF affected by the Tn5 insertion is marked in black and the genes coding for hypothetical proteins are marked in grey. The directions of the arrows represent the direction of the transcription. The complete genome of *D. solani* strain IPO2222 (GenBank accession: NZ_CP015137.1) was used to visualize the genetic organization.

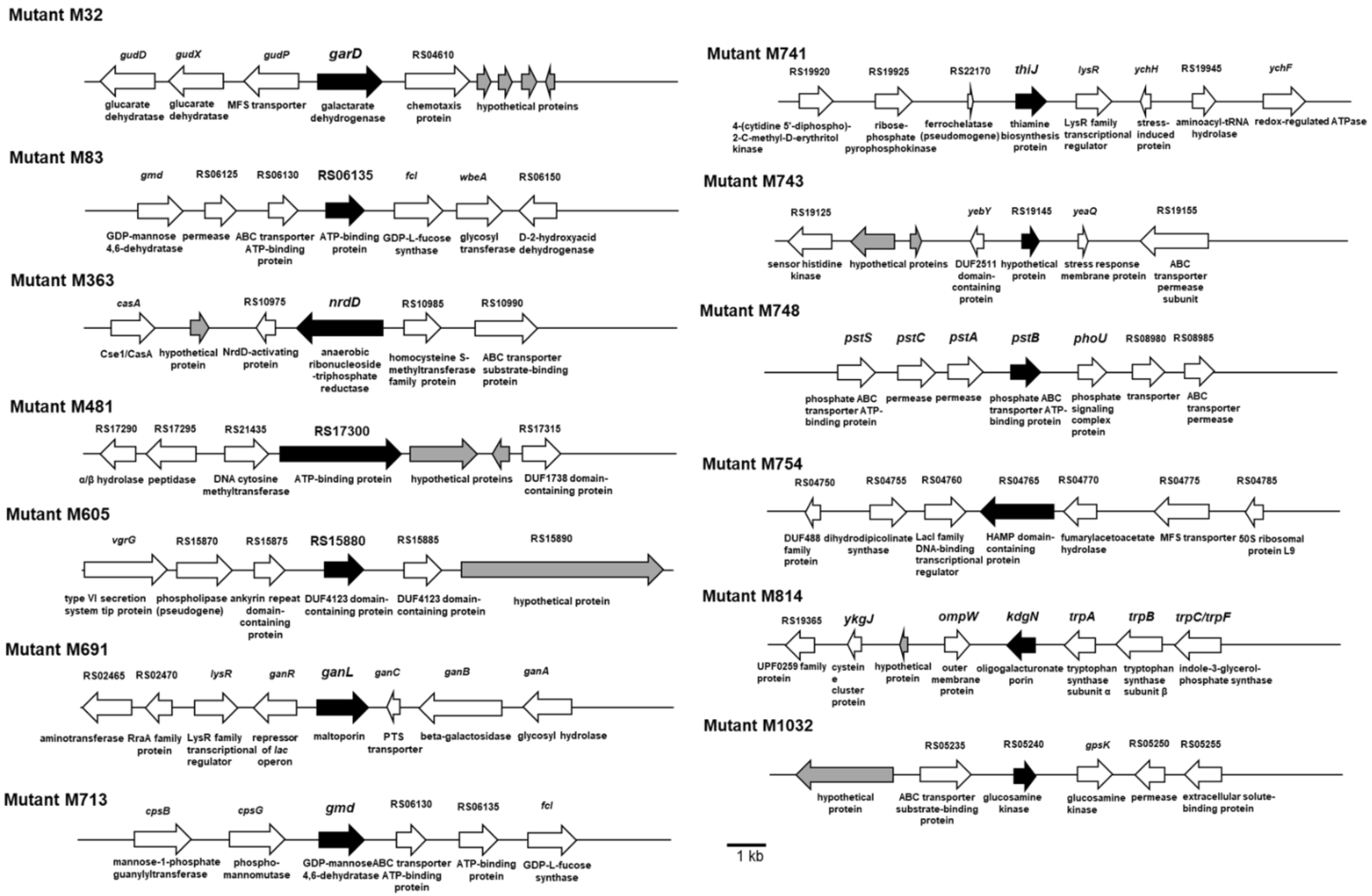

Supplementary Figure 2. Categories of proteins found in the extracts of *S. tuberosum* (green) and *S. dulcamara* (yellow)

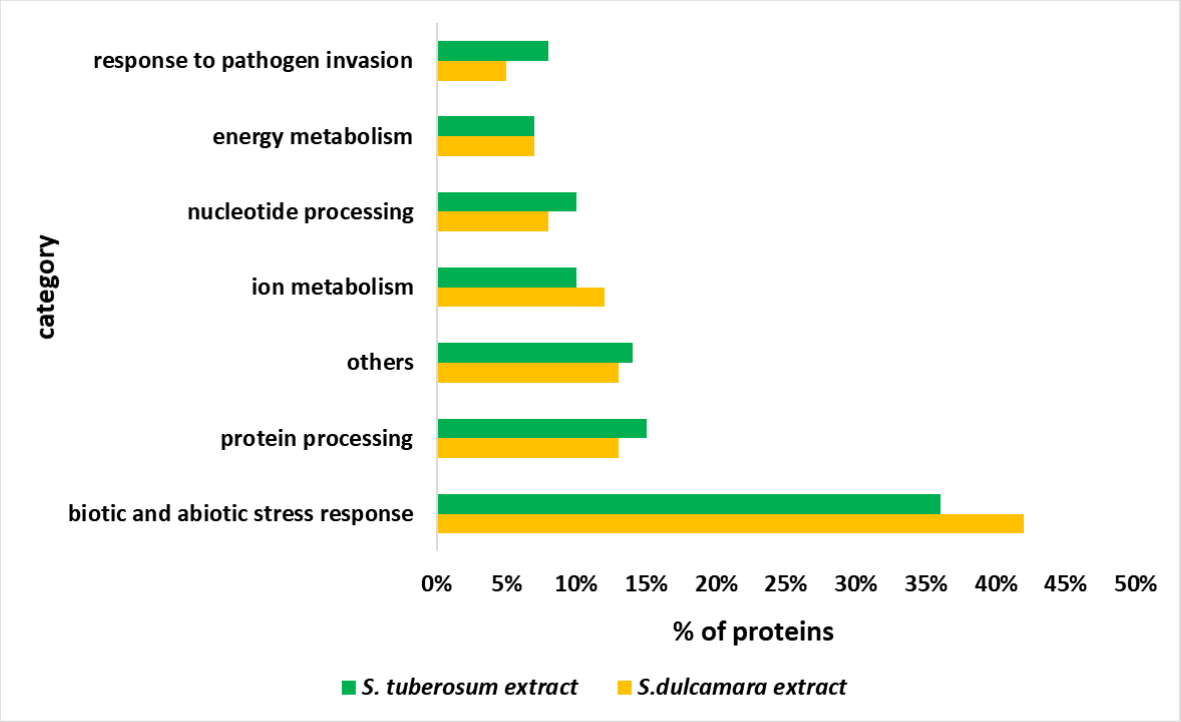

Supplement: Supplementary file 1 [file DataSheet_1.pdf]
